# DDX17 promotes the growth and metastasis of lung adenocarcinoma

**DOI:** 10.1038/s41420-022-01215-x

**Published:** 2022-10-22

**Authors:** Xiaohui Liu, Lu Li, Chengjie Geng, Shiyuan Wen, Cuiqiong Zhang, Chunmiao Deng, Xuejuan Gao, Gong Zhang, Qing-yu He, Langxia Liu

**Affiliations:** Key Laboratory of Functional Protein Research of Guangdong Higher Education Institutes and MOE Key Laboratory of Tumor Molecular Biology, Institute of Life and Health Engineering, Jinan University, Guangzhou, 510632 China

**Keywords:** Focal adhesion, Non-small-cell lung cancer, Cell invasion, Cell growth, Predictive markers

## Abstract

DEAD box RNA helicase 17 (DDX17) has been shown to be an RNA binding protein involved in RNA metabolism and associated with cancer progression. However, the biological role of DDX17 in the pathogenesis of lung adenocarcinoma (LUAD) has not been well characterized. Here, we demonstrated that DDX17 promoted the proliferation, migration and invasion of H1299 and A549 lung adenocarcinoma cells. Analyses of public datasets showed that DDX17 is upregulated in LUAD specimens. Our tumor xenograft models confirmed the in vivo promoting role of DDX17 in the growth and metastasis of LUAD. Mechanistic analyses further revealed that DDX17 protein interacts with the mRNA of MYL9 and MAGEA6 and upregulates their levels. MYL9 could mediate the function of DDX17 to regulate the actin cytoskeleton rearrangement and cell adhesion, particularly by modulating the stress fiber and focal adhesion formation, whereas DDX17 might inhibit the autophagy process through MAGEA6/AMPKα1 axis in LUAD cells. Collectively, our study revealed the oncogenic role and pathways of DDX17 in LUAD.

## Introduction

Lung cancer has the highest morbidity and mortality among all malignancies [1]. In clinical, non-small cell lung cancer (NSCLC), accounting for 80–85% of all lung cancer patients, are often diagnosed at an advanced stage of the disease [2]. NSCLC can be divided into three types: adenocarcinoma, squamous cell carcinoma, and large cell carcinoma. Among them, lung adenocarcinoma (LUAD) is the most common type, accounting for 70% of NSCLC cases. The main cause of deaths associated with the malignant cancers is their strong invasive feature. The vast majority of cancer-related deaths are attributed to the metastasis which is a multiple-step complex process, and lung cancer, especially NSCLC is among the most metastatic cancers [3, 4]. Despite considerable advances in medical research and technology, the prognosis of LUAD remains still poor [5]. Therefore, more efforts should be made to better understand the mechanisms of the pathophysiology of LUAD.

DEAD box helicase 17 (DDX17) belongs to the DEAD box family of ATP-dependent RNA helicases, and regulates RNA biosynthesis and metabolism [6, 7]. The DDX17 gene has two major mRNA isoforms resulting from the alternative translational initiation mechanism, these two mRNA isoforms encode respectively two protein isomers: p72 and p82, which are commonly co-expressed in cell lines and tissues and functionally undistinguished in the previous studies [8]. In addition to its important role in RNA processing, selective splicing, and biological processes of miRNA, DDX17 has been reported as a transcriptional co-regulator that is critical for various biological events such as proliferation, migration, apoptosis, and differentiation, and could be associated with cancer development [9–11]. DDX17 can activate estrogen receptor alpha (ERα), which is essential for ERα-responsive genes expression and breast cancer cell growth [12]. Upregulation of DDX17 enhances the gefitinib resistance in NSCLC via activation of β-catenin [13]. On the other hand, using TCGA and CPTAC data, we found that DDX17 was upregulated in LUAD specimens, hinting on the important role of DDX17 in the pathophysiology of LUAD. However, this hypothesis has not yet been verified experimentally in a comprehensive manner, especially, the role of DDX17 in cancer has never been tested in vivo in animal models, and little is known about the mechanistic basis of how DDX17 plays its roles in LUAD progression.

The present study used LUAD cell culture in vitro to examine the effect of DDX17 up- or down-regulation on cell proliferation, mobility, and ability of colony formation. Tumor xenograft models were then used to assess the function of DDX17 in tumor growth and metastasis in vivo. Based on the possible role of DDX17 on gene expression regulation reported previously in the literatures, we have performed high throughput transcriptomic sequencing by using lung adenocarcinoma H1299 LUAD cells with stable DDX17 knockdown. GO term and KEGG signaling pathway enrichment analyses of the differentially expression genes (DEGs) showed that DDX17 regulates cell adhesion and proliferation in H1299 cells. Two candidate genes, namely MYL9 and MAGEA6 were selected for further studies as the potential effectors of DDX17 protein, mediating respectively its functions in cytoskeleton remodeling and autophagy of lung adenocarcinoma cells. MYL9 is critically associated with actin stress fiber assembly and actomyosin contractility [14], and plays a crucial role in a variety of malignant tumors, such as breast cancer [15], colorectal cancer [16], bladder cancer [17], and NSCLC [18]. MAGEA genes have been considered to function as oncogenes whose high expression levels in multiple cancer lineages have been correlated with advanced stage and poor prognosis [19, 20]. Recent work shows that MAGEA6 drives ubiquitination and degradation of AMP-activated protein kinase (AMPK) by TRIM28 E3 ubiquitin ligase, coordinating cell growth, autophagy and metabolism [21–23].

## Results

### DDX17 is overexpressed in various cancers

**Fig. 1:**
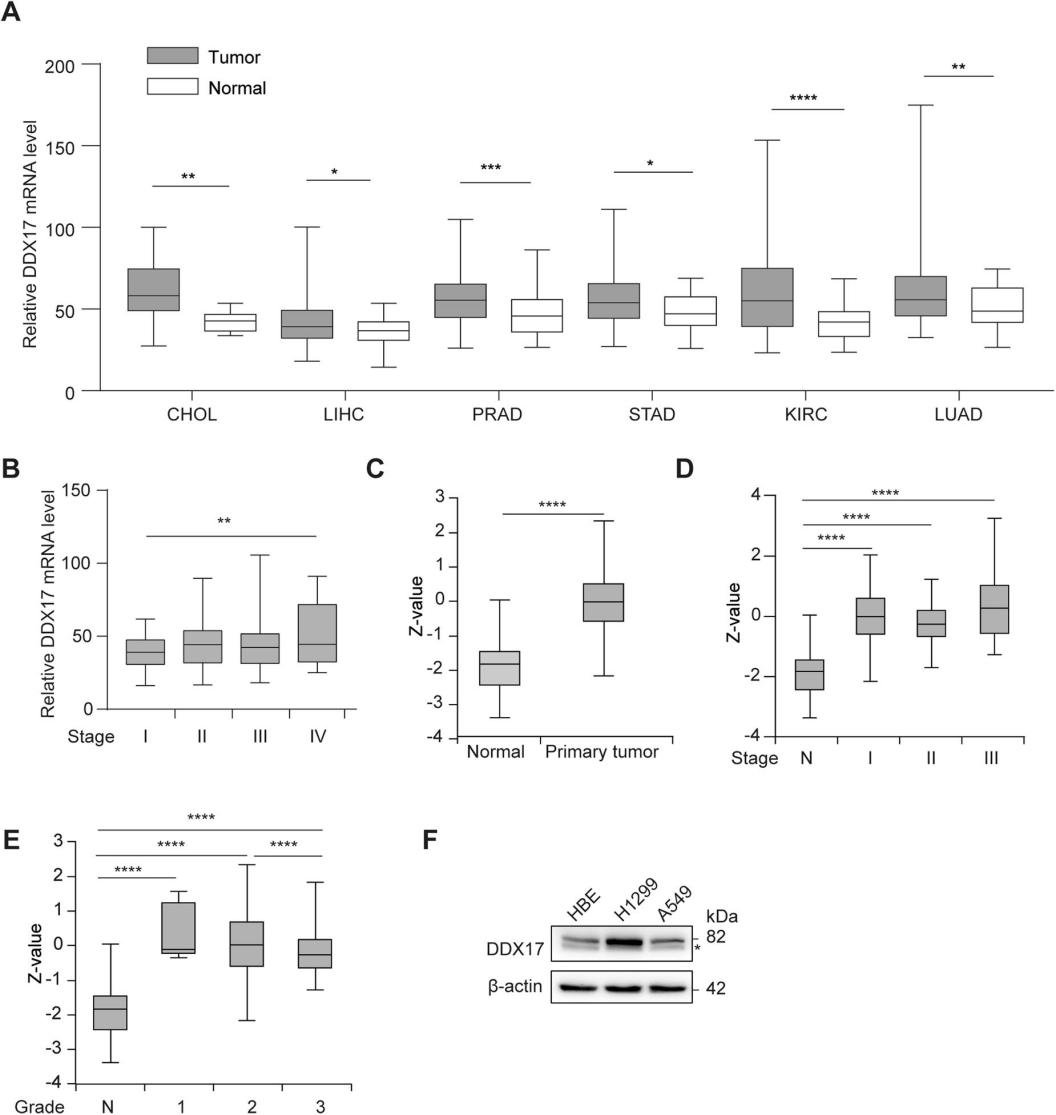
DDX17 is overexpressed in various cancers. **A** mRNA level of DDX17 in various tumor compared with the corresponding normal tissues from TCGA database. CHOL cholangiocarcinoma, LIHC Liver Hepatocellular Carcinoma, PRAD Prostate Adenocarcinoma, KIRC Kidney Renal Clear Cell Carcinoma, STAD Stomach Adenocarcinoma, LUAD Lung adenocarcinoma. **B** mRNA levels of DDX17 in various stages of LUAD (data from TCGA). **C**–**E** The expression of DDX17 protein in different stages (**C**, **D**) and grades (**E**) of lung adenocarcinoma and normal tissues analyzed by UALCAN cancer database. *Z*-values represent standard deviations from the median across samples for the given cancer type. Log2 Spectral count ratio values from CPTAC were first normalized within each sample profile, then normalized across samples. *p < 0.05, **p < 0.01, ***p < 0.001, ****p < 0.0001, determined by two-tailed Student’s *t*-tests (**A**, **C**) or two-way ANOVA (**B**, **D**, **E**). **F** DDX17 protein expression was analyzed by western blot in HBE, A549, H1299 cell lines. Asterisk (*) symbol indicates nonspecific binding.

We investigated the expression of DDX17 in the clinical samples from various cancer. The correlation between DDX17 expression and cancer patient was analyzed using RNA-seq data from TCGA project. DDX17 was significantly overexpressed in a wide variety of cancers (CHOL, LIHC, PRAD, STAD and KIRC) as compared to the corresponding healthy tissues at mRNA level. Especially, the mRNA expression level of DDX17 was markedly upregulated in LUAD tissues compared with that in normal tissues (Fig. 1A). We further assessed its possible association with the clinical pathological characteristics. As shown in Fig. 1B, there was a potent correlation between DDX17 expression and cancer stage progression. A higher expression of DDX17 protein was also detected in lung adenocarcinoma tissues using UALCAN cancer database, and the expression level of DDX17 was correlated with tumor stage and grade at varying degrees (Fig. 1C–E). However, prognosis using Kaplan-Meier survival analysis and performing univariate and multivariate Cox proportional hazards survival analysis indicated that DDX17 expression may not be an independent predictor of LUAD patients. Other risk factors might influence the results of prognosis analyses making their interpretations rather confusing (Fig. S1 and Table S1). We then further used Western blot to reveal that the expression of DDX17 in two commonly used LUAD cell lines (H1299 and A549) were also increased relative to the normal human bronchial epithelial (HBE) cell. These two LUAD cell lines were therefore used as cellular models in this study (Fig. 1F).

### DDX17 promotes LUAD cell growth and invasion in vitro and in vivo

**Fig. 2:**
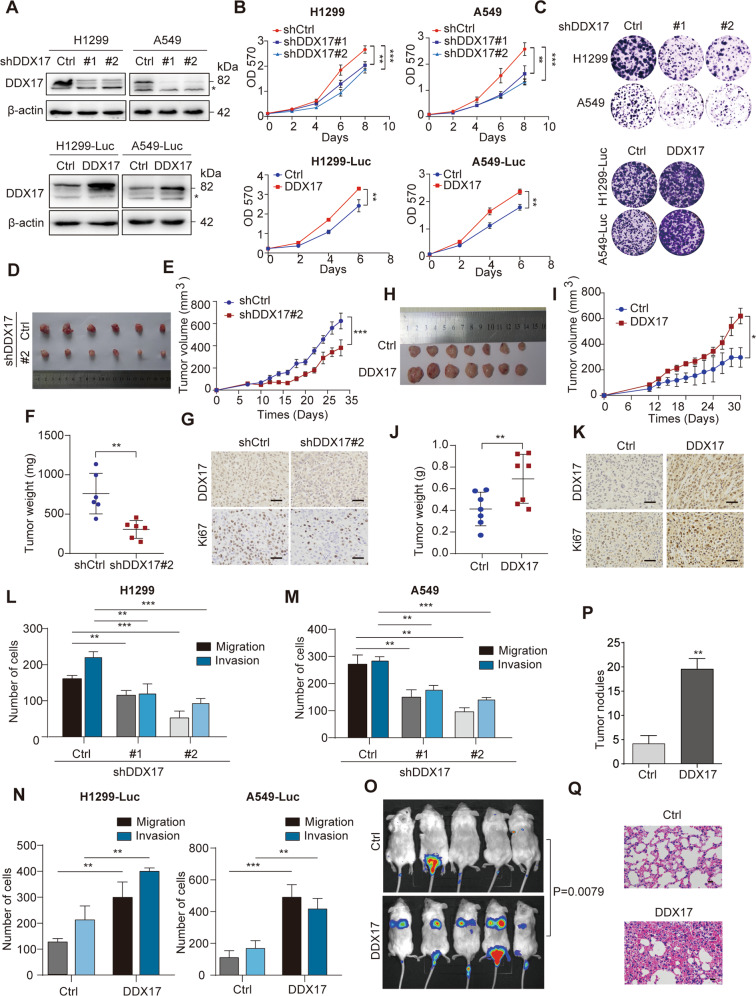
DDX17 promotes LUAD cell growth and invasion in vitro and in vivo. **A** DDX17 stable knockdown or overexpression in H1299 and A549 cells verified by western blot. **B** Cell proliferation determined by MTT assay. Data are presented as mean ± s.e.m. from three independent experiments. **p < 0.01; **p < 0.001, determined by two-way ANOVA. **C** Clone formation assays performed with indicated cells. **D** and **H** Image of tumors in nude mice injected with A549 cells bearing control shRNA or DDX17#2 shRNA (**D-G**, *n* = 6), or A549-Luc cells with stable DDX17 overexpression or corresponding control cells (**H**–**K**, *n* = 7). **E**, **I** Evolution of tumor volume measured at different time points in the indicated nude mouse groups. Data are presented as mean ± s.e.m. *p < 0.05, determined by two-way ANOVA; **F**, **J** statistical representation of subcutaneous tumor weight in the indicated nude mouse groups. Data are presented as mean ± s.e.m. **p < 0.01, determined by two-tailed Student’s *t*-tests; **G**, **K** representative images of Ki-67 IHC staining of the tumors from two groups. Scale bars = 20 μm. **L**, **M** Cell migration and invasion abilities analyzed by transwell assays in DDX17-knockdown H1299 (**L**) and A549 (**M**) cells as indicated. **N** Transwell migration and invasion assays were performed in H1299-Luc and A549-Luc with stable DDX17 overexpression, or control cells. Data are presented as mean ± s.d. from three independent experiments. **p < 0.01; **p < 0.001, determined by two-way ANOVA (**L**–**N**). **O** Bioluminescent images acquired at 18 days after tail intravenous injection of NCG mice with DDX17-overexpressing, or control H1299-Luc cells and corresponding quantification analyses (*n* = 5), *p-*values were determined by two-tailed Mann–Whitney *U* tests. **P**, **Q** Tumor nodule numbers (**P**) and HE staining (**Q**) of the lungs collected from the mice from the indicated groups. Scale bars = 20 μm. Data are presented as mean ± s.e.m. **p < 0.01, determined by two-tailed Student’s *t*-tests.

In order to investigate the function of DDX17 in LUAD, we generated stable DDX17 knockdown H1299 and A549 cell lines using recombinant lentiviral shRNA expression system. Two shRNAs (shDDX17#1 and shDDX17#2) were revealed to be efficient with >70% reduction at protein level in the respective stable cell lines (Fig. 2A, upper panels). Meanwhile, stable DDX17 overexpressing or control LUAD cell lines, including H1299 Luc-Ctrl, H1299 Luc-DDX17, A549 Luc-Ctrl, A549 Luc-DDX17 were also generated as described in Materials and methods section. Western blot confirmed the exogenous DDX17 expression in these cells (Fig. 2A, lower panels). We then performed MTT and Clone formation assays using these cells to assess the function of DDX17 in LUAD cell proliferation. The results showed that DDX17 knockdown significantly decreased the cell proliferation (Fig. 2B, upper panels) and cell clone formation (Fig. 2C, upper panels), whereas DDX17 overexpression significantly increased these cellular functions proliferation and cell clone formation in vitro (Fig. 2B, C, lower panels). To further confirm the function of DDX17 in LUAD growth, the hereinabove A549 cells with stable DDX17 silencing or overexpression, in parallel with their respective control cells, were injected into the dorsal flank of nude mice. Twenty-eight days after injection, we found that the tumors formed by DDX17-deficient A549 cells were generally smaller than those in the control group (Fig. 2D), with significantly delayed tumor growth compared with the latters (Fig. 2E). Accordingly, the average weight of tumors formed in DDX17 knockdown group was obviously inferior to that in the control group (Fig. 2F). Moreover, DDX17-knockdown reduced the number of Ki-67 positive tumor cells in the tumor xenografts of nude mice (Fig. 2G). As expected, opposite effects have been observed in mice injected with cells overexpressing DDX17 (Fig. 2H–K). These results confirmed the promoting function of DDX17 on LUAD growth in vitro and in vivo.

We then examined the role of DDX17 in the motility of LUAD cancer cells. Transwell migration and matrigel invasion assays were performed using the same stable cell lines with DDX17 knockdown or overexpression as described in above. The results indicated that DDX17 knockdown in H1299 and A549 cells resulted in a significantly decreased level of cell migration and invasion in vitro (Fig. 2L, M, Fig. S2A, B). Conversely, DDX17 overexpression in H1299-Luc and A549-Luc cells clearly increased cell migration and the ability to invade through the extracellular matrix coating (Fig. 2N, Fig. S2C, D). Again, in vivo experiments have been subsequently carried out to confirm these results. H1299-Luc cells stably overexpressing DDX17 or control cells were intravenously injected into the tail veins of NCG mice and the metastatic ability of these cells were evaluated using luciferase-based bioluminescence tracing. As showing in Fig. 2O, H1299-Luc cells overexpressing DDX17 displayed a superior ability to metastasize to the lung than the control cells, as reflected by the stronger bioluminescence signals in the lung. Meanwhile, a significant increase in terms of tumor nodule number was also observed in the mice injected with the cells overexpressing DDX17 as compared with the control group (Fig. 2P), which was also evidenced by histological examination of the lung (Fig. 2Q). Together, these results indicated that DDX17 can significantly enhance the migration and metastasis of LUAD cells in vitro and in vivo.

### DDX17 upregulates the expression of MYL9 and MAGEA6 in LUAD cells

**Fig. 3:**
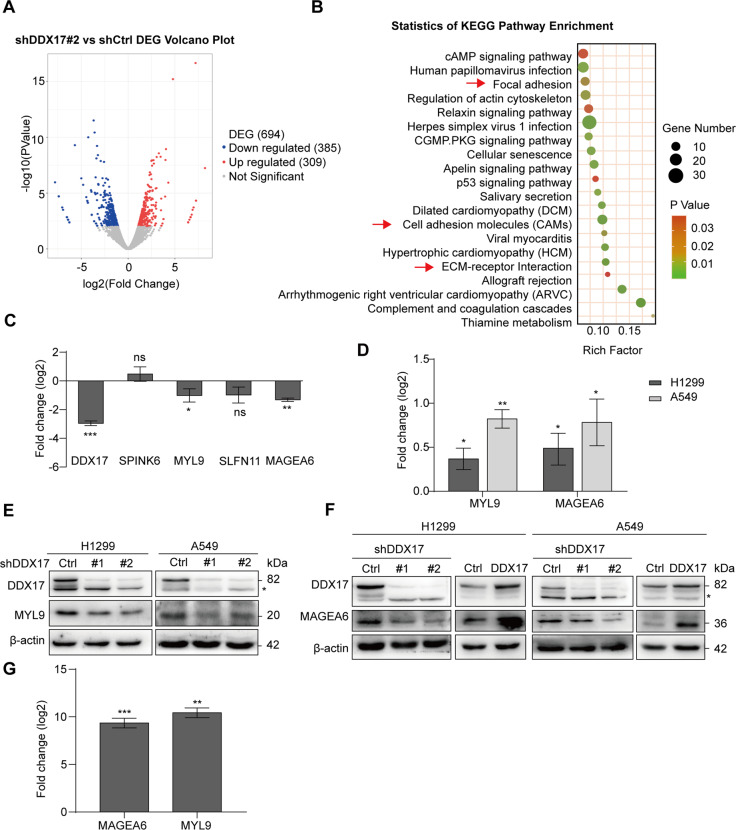
DDX17 regulates the mRNA and protein levels of MYL9 and MAGEA6. **A** Volcano plot of global DEGs between H1299-shDDX17#2 cells and their corresponding control cells. **B** the functions of DEGs were predicted by analysis of KEGG by DAVID (https://david.ncifcrf.gov/). **C** The log2 transformation of the fold-change of the relative mRNA expression levels in the 4 DEGs selected for qRT-PCR in DDX17 silencing or control cells. **D** mRNA level of MYL9 and MAGEA6 in H1299 and A549 cells transiently transfected with Flag-DDX17 plasmids or Flag-vector. **E**, **F** DDX17 and MAGEA6 expression in the indicated cells assessed by western blotting. β-actin was used as loading control. **G** The relative abundance of MYL9 and MAGEA6 mRNA present in the DDX17-IP assay by qRT-PCR. Data are presented as mean ± s.e.m. (**C**, **D**, **G**). *p < 0.05, **p < 0.01, ***p < 0.001, determined by two-tailed Student’s *t*-tests.

In order to explore the underlying mechanism of DDX17-mediated LUAD progression. RNA-sequencing was performed to identify differentially expressed genes (DEGs) in H1299 cells upon stable DDX17 silencing. The sequencing data showed that the RPKM density distribution of shDDX17 cells and control cells was highly similar (Fig. S3A), with respectively 83.6% (control group) and 82.96% (shDDX17 group) of reads mapped to the RefSeq (Fig. S3B). Pearson’s correlation coefficient between the two groups was 0.98 (Fig. S3C), indicating that the sequencing results were valid and reliable. A total of 694 DEGs were identified, including 309 upregulated genes and 385 down-regulated genes (Fig. 3A). Functional enrichment analysis by GO terms showed that these genes participated mainly in cell adhesion, regulation of cellular process, and regulation of cell proliferation (Fig. S3D). Similar results were also obtained by KEGG enrichment analysis, showing that the DEGs were involved in regulation of actin cytoskeleton, cell adhesion molecules (CAMs), and ECM-receptor interaction (Fig. 3B). We then verified the sequencing results of the top ranked DEGs (SPINK6, MYL9, SLFN11, and MAGEA6) by RT-qPCR. The results showed that the mRNA levels of MYL9 and MAGEA6 were most significantly decreased in DDX17 knockdown cells (Fig. 3C). Vice versa, DDX17 overexpression in the cells significantly increased the mRNA levels of these two genes (Fig. 3D). In consistence with the mRNA expression level, protein level of MYL9 and MAGEA6 in both H1299 and A549 cells were also confirmed to be upregulated by DDX17 (Fig. 3E, F), indicating that DDX17 upregulated of MYL9 and MAGEA6 expression levels. RIP assays using H1299 cell lysates further demonstrated that MYL9 and MAGEA6 mRNA could be specifically enriched using an anti-DDX17 antibody (Fig. 3G), suggesting that DDX17 bound to the mRNA of MYL9 and MAGEA6. Next, we examined whether DDX17 knockdown affected the stability of MAGEA6 and MYL9 mRNA. As shown in Fig. S4, DDX17 knockdown did not seem to affect the rate of decay of MAGEA6 or MYL9 mRNA in cells under actinomycin D treatment. Taken together, these results suggested that DDX17 might regulate the expression of MYL9 and MAGEA6 through a post-transcriptional mechanism other than the stabilization of mRNA.

### DDX17 regulates cell adhesion and cytoskeleton reorganization of LUAD cells via its target MYL9

**Fig. 4:**
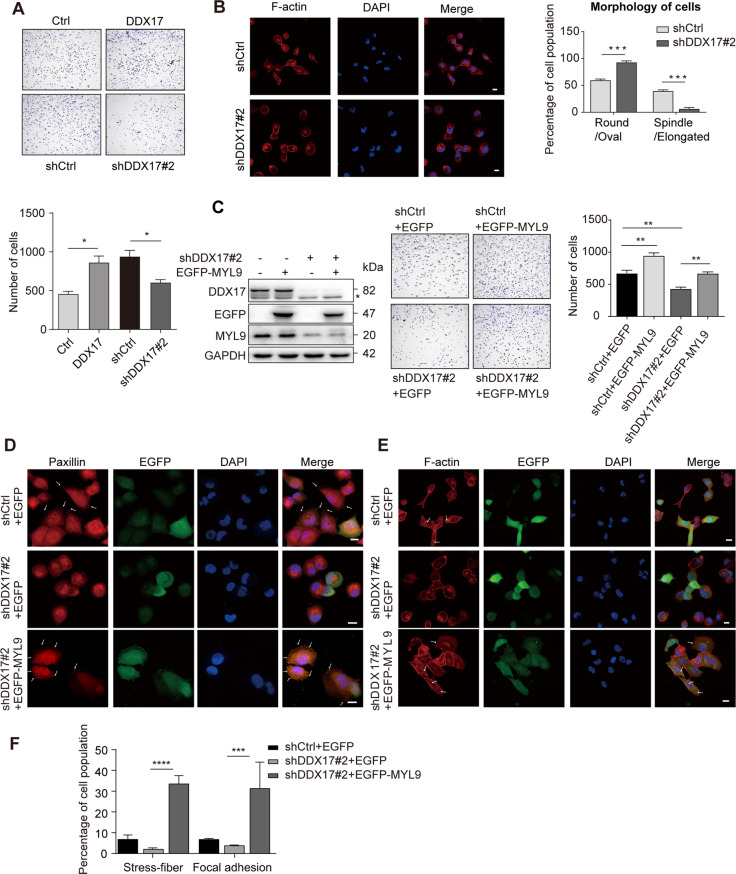
DDX17 regulates cell adhesion and cytoskeleton reorganization of LUAD cells via its target MYL9. **A** Cell adhesion assays of DDX17-knockdown or overexpressing H1299 cells and corresponding control cells. Representative images (top) of the cells and cell counting results (bottom) were shown, and data are presented as mean ± s.e.m. from three independent experiments. *p < 0.05, determined by two-tailed Student’s *t*-tests. **B** F-actin cytoskeleton in shCtrl and shDDX17#2 H1299 cells was monitored by rhodamine-phalloidin staining. Nuclei were counterstained with DAPI (left panel). Scale bars: 10 μm. Right pane: Cells with various morphology features were counted, (mean ± s.e.m. of counted cells, *n* ≥ 30 cells per cohort, determined by two-tailed Student’s *t*-tests). **C** H1299-shDDX17#2 cells or control cells were transiently transfected with EGFP-MYL9 or control plasmids, and analyzed by western blotting with appropriate antibodies (left); representative photos of cell adhesion assays with the indicated cells (middle) and the corresponding quantification analyses, and data are presented as mean ± s.e.m. from three independent experiments. **p < 0.01, determined by two-way ANOVA (right). **D** Representative images of IF staining of Paxillin of the indicated cells. **E** F-actin cytoskeleton staining using rhodamine-phalloidin. Scale bars: 10 μm. **F** Quantification analysis of stress fiber and FAs formation. Data are presented as mean ± s.e.m. from three independent experiments. ***p < 0.001, determined by two-way ANOVA.

Our transcriptome analysis suggested that DDX17 is involved in the regulation of cell adhesion and actin cytoskeleton. To confirm these results, cell adhesion and IF assay were performed with H1299 cells. As shown in Fig. 4A, DDX17 overexpression significantly promoted the adhesion of H1299 on matrigel basement membrane matrix (upper panels), whereas inhibition of DDX17 expression inhibited the adhesion of these cells (lower panels). Interestingly, IF staining showed that DDX17 knockdown resulted in obvious changes of the morphology and actin cytoskeleton of H1299 cells. Whereas the control H1299 cells (shCtrl cells) were generally in spreading form of cobblestone, and displayed structures resembling the stress fibers stained by Phalloidin in the cytoplasm, shDDX17 cells were generally in more contracted round or oval shape, with F-actin more concentrated at the plasma membrane, forming a ring-like structure (Fig. 4B). Based on previous reports that MYL9 is involved in cytoskeletal events including formation of stress fiber and focal contact networks [14, 18, 24], the role of MYL9 in the regulation of cell adhesion and cytoskeletal reorganization by DDX17 was subsequently investigated. shCtrl or shDDX17 H1299 cells were transfected either with EGFP-MYL9 or control plasmids for 24 h. MYL9 overexpression in both shRNA and shDDX17 cells was first confirmed by western blot (Fig. 4C, left). Cell adhesion assay showed that MYL9 overexpression restored the adhesion ability of DDX17-deficient cells (Fig. 4C, right). Paxillin is a focal adhesion (FA) associated protein serving as a scaffold protein at focal adhesions required for cell attachment, spreading, and migration. It is commonly used as a FA marker in cell immunofluorescence experiments [25]. Therefore, in order to evaluate the possible role of DDX17 in FA formation, we have performed cell immunofluorescence experiments with an anti-Paxillin antibody in shCtrl or shDDX17 H1299 cells transfected with EGFP-MYL9 or control plasmids. As shown in Fig. 4D, in control cells, scattered patch form structures (denoted by arrows) stained by anti-Paxillin antibody could be observed at the spreading edge of cells, but not in the round shape shDDX17 cells where Paxillin was obviously more concentrated in the perinuclear region. Interestingly, the overexpression of MYL9 in DDX17-deficient cells induced, in addition to the morphological change from the round and contracted shape to the more extended polygonal form, a drastic increase in density of FAs labeled by Paxillin staining. Moreover, EGFP-MYL9 was also found in co-localization with Paxillin in these FAs. Formation of stress fibers is essential for cell movement and migration [14]. Consistently, overexpression of MYL9 in DDX17-deficient cells also promoted stress fiber formation (Fig. 4E, F, denoted by arrows). Based on these results, we speculated that MYL9 could be an effector of DDX17 in the regulation of cell motility of LUAD cells.

### DDX17/MAGEA6/AMPKα1 signaling regulates LUAD cell growth by autophagy

**Fig. 5:**
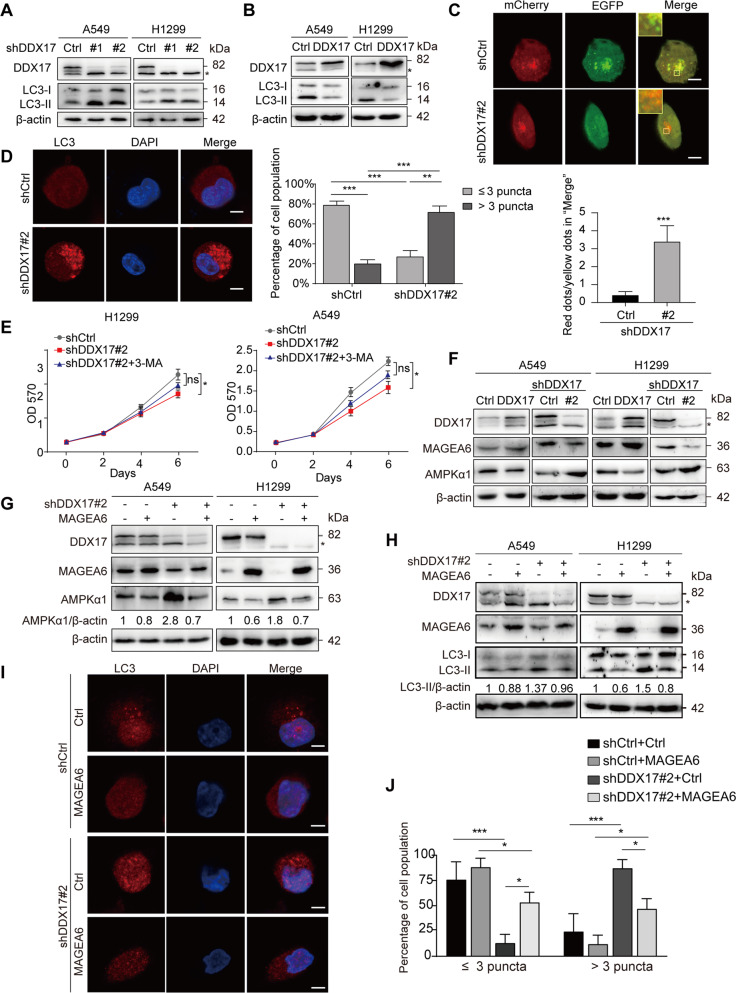
DDX17/ MAGEA6/AMPKα1 signaling regulates autophagy in LUAD cells. **A**, **B** Level of LC3 protein by western blotting in DDX17-knockdown cells (**A**), or DDX17-overexpression cells (**B**), and the corresponding control cells**. C** H1299-shDDX17#2 cells or control cells were transiently transfected with mCherry-EGFP-LC3 reporter plasmids, and analyzed by IF assays. Yellow signals represented the non-acidic autophagosomes and red signals represented the acidic autophagolysosomes in merged images (Top panels). Scale bars = 10 μm. Bottom panels: quantitative analysis of red dots / yellow dots of the merged images in cells (mean ± s.e.m. of counted cells, *n* ≥ 12 cells per cohort, determined by two-tailed Student’s *t*-tests). **D** Representative photos of IF staining of LC3 (red) puncta in H1299-shDDX17#2 cells or control cells (left panel). Scale bars =10 μm. The right panel is the statistical analysis of LC3 puncta (mean ± s.d. of counted cells, *n*  ≥ 12 cells per cohort, determined by two-way ANOVA). **E** MTT assays were performed in DDX17 silencing or control H1299 cells (left) treated with or without 3-MA (1 mM), and A549 cells (right) treated with or without 3-MA (0.25 mM), respectively. Data are presented as mean ± s.e.m. from three independent experiments. *p < 0.05; ns, *p* > 0.05, determined by two-way ANOVA. **F** Western blotting showing the level of MAGEA6 and AMPKα1 in DDX17-knockdown cells, or DDX17-overexpression cells. **G** AMPKα1 level in H1299-shDDX17 #2 cells or control cells with or without MAGEA6 stable overexpression. **H** LC3 levels in H1299-shDDX17 #2 cells or control cells with or without MAGEA6 stable overexpression. **I** Representative photos of IF staining of LC3 (red) puncta in H1299-shDDX17 #2 cells or control cells with or without MAGEA6 stable overexpression. **J** Statistical analysis of LC3 puncta in **I** (mean ± s.e.m. of counted cells, *n* ≥ 12 cells per cohort, determined by two-way ANOVA).

Accumulating evidence indicates that autophagy plays critical roles in the development, maintenance, and progression of LUAD [26–28]. Overexpression of DDX17 enhances malignant migration and invasion of glioma cells by repressing beclin1 expression [29]. Zhang et al. reported that DDX17 is important for the autophagy regulatory network through clinical data analyses of diverse stages for colorectal cancer [30]. Based on these reports, we investigated whether DDX17 regulates autophagy process in lung adenocarcinoma cells. We first determined whether DDX17 regulated the expression level of LC3-II, a common autophagy marker located in the autophagosomes. As shown in Fig. 5A, B, DDX17 knockdown increased the expression of LC3-II, whereas DDX17 overexpression reduced the accumulation of LC3-II in both A549 and H1299 cells. Next, confocal fluorescence microscopy using mCherry-EGFP-LC3 reporter showed that knockdown of DDX17 remarkably promoted the autophagy flux, reflected by a strong increase of the number ratio of the red dots and the yellow dots (Fig. 5C). This observation was subsequently confirmed by the endogenous LC3 staining in wild type and DDX17 knocked down cells. As shown in Fig. 5D, mature autophagosomes represented by the LC3 positive puncta were massively present in the cytoplasm of the control cells, but were absent in DDX17 knocked down cells. These results confirmed the critical role of DDX17 in the autophagy process of the cell. In order to evaluate the impact of this function of DDX17 on LUAD development, we used an autophagy inhibitor 3-methyladenine (3-MA) to treat the DDX17-deficient cells and found that this could partially restore the cell viability and colony-forming capacity of DDX17-deficient cells (Fig. 5E and Fig. S5). Collectively, these results suggest that DDX17 inhibited autophagy to promote tumor progression.

It has been reported that AMP-activated protein kinase (AMPK) α1, a bio-energy sensor responsible for maintaining cellular energy homeostasis in all eukaryotic cells, is ubiquitinated and degraded by MAGEA6-TRIM28, thereby inhibiting autophagy [31]. This prompted us to investigate whether DDX17 regulates cell autophagy through MAGEA6/AMPK axis in LUAD cells. As shown in Fig. 5F, overexpression of DDX17 upregulated the level of MAGEA6 protein and downregulated AMPKα1 protein in H1299 and A549 cells. On the contrary, MAGEA6 protein levels were significantly decreased in shDDX17#2 cells, while AMPKα1 was accordingly upregulated. Then, we stably overexpressed MAGEA6 in A549-shDDX17#2 and H1299-shDDX17#2 cells and examined whether the overexpression of MAGEA6 could counteract the effect of DDX17 knockdown. It turned out that the protein levels of AMPKα1 and LC3-II as well as the number of LC3 positive puncta in MAGEA6-overexpressing cells were virtually identical to that of control cells without DDX17 deficiency, as demonstrated by western blotting (Fig. 5G, H) and immunofluorescence staining (Fig. 5I, J) assays. Taken together, these data suggest that DDX17 might regulate autophagy in LUAD cells through MAGEA6/AMPKα1 axis.

## Discussion

DEAD/DEXH box helicases play a universal role in RNA metabolism, including transcription, alternative splicing and miRNA processing, which are often dysregulated in many malignant cancers. While controversies regarding the exact role played by DDX17 in diverse cancers have been reported or suggested [8, 11, 13, 32–34], the biological function of DDX17 in LUAD has not yet been investigated by systematic approaches. Meanwhile, the heterogeneities characteristic to this type of cancer implying the complexity and originality of its underlying mechanisms require the investigation of the exact role of DDX17. Combining in vitro and in vivo approaches, our present study clearly indicates that DDX17 plays a promoting role in the progression and metastasis of LUAD, providing new data for the confirmation of DDX17 as a pro-oncogenic gene and a potential target for the therapy of LUAD. Specifically, we found that knockdown of DDX17 significantly reduced cell proliferation and invasion, while its overexpression enhanced these two functions. Importantly, as the first in vivo study of DDX17’s functions in cancer, our xenograft experiments in mice clearly indicated that DDX17 plays a pro-oncogenic role in LUAD by promoting both tumor growth and metastasis.

As one of the most studied programmed cell death processes, autophagy has since longtime been shown to play critical roles in the genesis and development of cancer. Autophagy is a double-edged sword in carcinogenesis. Both pro-tumor and antitumor functions of autophagy have been reported, which largely dependent on the specific genetic context and tumor stage [35]. Autophagy can be tumor promoting by maintaining cell synthesis pathways and energy balance [28, 36, 37]. Alternatively, it can be tumor suppressive through facilitating the degradation of oncogenic molecules, or reducing genome mutations that drive tumorigenesis [38, 39]. In addition, it has been reported that autophagy limits inflammation and tissue damage that can promote the initiation of cancer, suggesting that autophagy is important for the suppression of spontaneous tumorigenesis [35, 40]. Interestingly, one of the candidate effectors of DDX17 protein that we identified in this study was MAGEA6, which is not only associated with cancer but also involved in the degradation of AMPK [31]. AMPK-mTOR pathway is well known for its role in modulating autophagy in a variety of different cancers [22, 23, 31, 35]. Our converging results tend to depict a novel DDX17/MAGEA6/AMPK regulatory axis inhibiting the autophagy process in LUAD cells and facilitating cellular proliferation and the growth of LUAD in vivo. Particularly, our study indicated that shDDX17 LUAD cells exhibited elevated AMPKα1 and autophagy levels, suggesting that the suppression of cell growth owing to DDX17 deficiency may be attributed to the increase of autophagy level in these cells. Further exploration of this finding could be valuable for cancer prevention and treatment.

Cell motility is closely related to the coordinated regulation of cell adhesion turnover and actin cytoskeletal dynamics. We have demonstrated that DDX17 promoted LUAD cell metastasis, which had not been shown before. Moreover, DDX17 knockdown decreasing MYL9 expression led to the morphological change with reduced focal adhesions and stress fiber formation in LUAD cells. It has been previously reported that MYL9 promoted the actin-myosin contraction, which strengthened the tension of FAs, thereby promoting movement [14, 41–43]. In our study, ectopic expression of MYL9 significantly enhanced the formation of stress fibers and focal adhesions in DDX17-deficient LUAD cells, suggesting that MYL9 might indeed mediate the function of DDX17 to regulate the actin cytoskeleton and adhesion of LUAD cells. Our results are reminiscent of the bioinformatics analysis reported by Tan et al. in NSCLC cell models. They showed that MYL9 expression level was significantly increased in late stages (stages III and IV) of NSCLC, and expression level of MYL9 in NSCLC with lymphatic metastasis was significantly higher than that in NSCLC without lymphatic metastasis, thus suggesting that MYL9 may also be involved in NSCLC metastasis [18]. A recent report performed in several colorectal cancer cell lines also showed that MYL9 overexpression promoted cell proliferation, invasion, migration and angiogenesis [41]. These although preliminary results seem to point out an important role of MYL9 in cancer development and further investigation should be promising for the better understanding of cancer pathophysiology.

Apart from its role as a co-factor of various transcription factors, DDX17 has also been shown to be involved in mRNA processing and maturation. We found in this study that DDX17 bound to the mRNAs of MAGEA6 and MYL9 and increased their levels. This might hint on a novel post-transcriptional mechanism of mRNA regulation by DDX17. Further studies aiming to determine the precise structure-function relationship would be necessary to confirm and explore this finding.

Taken together, the present study combining in vitro and in vivo approaches has confirmed the critical role of DDX17 protein in the growth and metastasis of LUAD. Mechanistic investigations revealed two target genes of DDX17 protein, namely MAGEA6 and MYL9 as its effectors to mediate respectively its functions in cell autophagy and actin skeleton remodeling.

## Materials and methods

### Cell culture and vectors

HEK293 cells, human lung cancer cell lines H1299 and A549 (American Type Culture Collection, Rockville, MD, USA) were cultured in DMEM medium (Life Technologies, Gaithersburg, MD, USA) with 10% fetal bovine serum (Life Technologies) at 37 °C in 5% CO_2_. All cells were free of mycoplasma contamination by TransDetect PCR Mycoplasma Detection Kit (Transgen, Beijing, China).

pCMV-N-Flag-DDX17, pEGFP-N1-MYL9, and pLVX-IRES-EGFP-BSD-MAGEA6 were constructed as previously described [24]. All plasmids were sequenced and confirmed for accuracy. The lentiviral shRNA vectors against DDX17 (constructed with pLKO.1 vector), and the corresponding control shRNA vector were obtained from Sigma (Sigma Aldrich, St. Louis, MO, USA). The sequences were as follows: shDDX17#1, 5′- CCGGGCACAGAAGAAACATGGCAAACTCGAGTTTGCCATGTTTCTTCTGTGCTTTTTG-3′; shDDX17#2, 5′- CCGGGCAGAGGATTTCCTTCGTGATCTCGAGATCACGAAGGAAATCCTCTGCTTTTTG-3′; and control shRNA, 5′-UUUGUACUACACAAAAGUACUG-3′.

### Western blot analysis

Western-blot assays were used to analyze protein expression [44]. The following antibodies were used: DDX17 (Proteintech, Cat. 19910-1-AP), MAGEA6 (SAB, Cat. 43096), p62 (Proteintech, Cat. 18420-1-AP), LC3 (Sigma, Cat. L8918), β-actin (Proteintech, Cat. 81115-1-RR), GAPDH (Proteintech, Cat. 10494-1-AP), AMPKα1 (Abcam, Cat. ab32047), paxillin (Proteintech, Cat. 10029-1-Ig), GFP (Proteintech, Cat. 66002-1-Ig), MYL9 (Abcam, Cat. ab191393) and the HRP-conjugated goat anti-rabbit/mouse secondary antibody (Jackson ImmunoResearch, PA, USA).

### Transduction

HEK293 cells at 70–80% confluence were transiently transfected with the appropriate lentiviral expression vectors using Lipofectamine 2000 (Life technologies corporation Gaithersburg) according to the manufacturer’s instructions. 4 μg of the target plasmids, 2 μg of psPAX2 plasmids and 1 μg of pMD2.G plasmids were used for each transfection. 48 h after transfection, supernatant containing viral particles was collected and filtered. Target cells were infected for 48 h with filtrated viral supernatant, and then selected using 2 μg/mL puromycin or 3 μg/mL blasticidin. The luciferase-expressing cell lines H1299-Luc and A549-Luc were generated by lentiviral particles of luciferase plasmid. And pLVX overexpression plasmid or shRNA were used to establish cells constitutively expressing or repressing DDX17.

### The Cancer Genome Atlas (TCGA), Clinical Proteomic Tumor Analysis Consortium (CPTAC) data analysis

The data of DDX17 were extracted and analyzed from the publicly available RNA-Seq data of mRNA level via the TCGA data portal (https://portal.gdc.cancer.gov/projects/TCGA-LUAD). Gene expression data from RNA-Seq results were quantified by FPKM. The relationship between DDX17 protein level and clinical pathological characteristics, and the transcription levels of DDX17 in other types of cancer tissues were analyzed using UALCAN (http://ualcan.path.uab.edu), which is a comprehensive, user-friendly, and interactive web resource for analyzing canceromics data (TCGA, MET500 and CPTAC).

### Cell viability assay

Cell proliferation was assessed using MTT assay. Briefly, the cells were seeded into 96-well plates at a density of 1000 cells/well. The plates incubated with MTT at 37 °C for 4 h and then were exposed to DMSO for 10 min. The cell viability was evaluated by checking the absorbance at 570 nm.

### Colony formation assay

H1299 or A549 cells were seeded in 6-well plates at a density of 1000 cells per well for 14 days. Colony formation was performed as previously described [45].

### Transwell assay

Transwell assays were conducted to evaluate the cell migration and invasion using the HTS transwell-24 system (Corning, NY, USA), which is an array of 24 individual Boyden chambers with 8-μm pore size transwell membranes. Cell migration assays were performed as previously described [45]. For invasion assays, the upper chambers were coated with matrigel. Cells on the lower surface were fixed, stained and counted at 8–12 h after seeding.

### Immunofluorescence assays

Immunofluorescence (IF) assay was performed to determine the subcellular localization of the indicated proteins as described previously [46].

### Tumorigenicity in nude mice

Male BALB/c nude mice (Nanjing Biomedical Research Institute of Nanjing University, NBRI) aged 6–8 weeks (*n* = 6–7/group, random grouping) were fed in Laboratory Animal Research Center of Jinan University. The experimental was carried out in the specific pathogen-free environment in Laboratory Animal Center of Jinan University. Each experiment was carried out in line with specific protocols as well as internal biosafety and bioethics guidelines from Laboratory Animal Ethics Committee of Jinan University (20210705-13). Approximately 5 × 10^6^ A549 cells in equal volumes of PBS and Matrigel were subcutaneously injected into the flanks of mice to establish tumor xenografts. Tumor size was measured using calipers every 2 days, and tumor volume was calculated according to the equation: *V* = (length × width^2^)/2. After 4 weeks, each mouse was performed cervical dislocation, and the tumors were collected for western blot and histological analysis.

### In vivo tumor metastatic assay

In vivo tumor metastasis assays were performed by the tail vein injection of H1299 cells. Luciferase-expressing H1299-Luc cells (1 × 10^6^ cells) were injected into male nude NOD-Prkdc^em26Cd52^Il2rg^em26Cd22^ (NCG) mice aged 6–8 weeks (Nanjing Biomedical Research Institute of Nanjing University, NBRI) via the tail vein (*n* = 5 /group, random grouping). Metastasis was monitored 18 days after tail intravenous injection using bioluminescence imaging by IVIS Spectrum in vivo imaging system (PerkinElmer, Waltham, MA, USA). Lung metastasis was examined by routine histopathological analysis. All animal experiments were approved by the committee on the use of live animals in Jinan University.

### RNA extraction, library preparation, and RNA-Seq

Total RNA was extracted from H1299 cells using TRIzol® Reagent (Invitrogen, Carlsbad, CA, USA) according to the manufacturer’s instructions. Then, the RNA integrity, concentration and purity were evaluated by using an Agilent 2100 Bioanalyzer and RNA Nano 6000 assay kit (Agilent Technologies, Santa Clara, CA, USA). Sequencing libraries were established using a VAHTS™ mRNA-seq V3 library prep kit for Illumina® (Vazyme, Nanjing, China) according to the manufacturer’s protocol. For RNA-seq assay, DNase I was added to eliminate DNA contamination. Illumina sequencing was performed at Vazyme Biotech Co., Ltd. (Nanjing, China). In short, mRNA was enriched by magnetic beads with Oligo (dT), and then randomly digested into short fragments about 200 bp. First-strand cDNA was generated from mRNA fragments and then double-stranded DNA was synthesized in the presence of DNA Polymerase I and RNase H. The double-stranded cDNA was purified with VAHTS™ DNA clean beads (Vazyme, China), followed by end-repair, adenylation and adapter ligation. The library was amplified by PCR amplification, then sequenced on an Illumina HiSeq X Ten sequencer at paired-end mode (PE150).

### Identification of differentially expression genes (DEGs)

The sequenced reads from Illumina HiSeq X were performed to check the quality by using FastQC. EdgeR software package was used for gene differential expression analysis of paired samples [47]. The absolute value of log2 (Fold change) > 1 and the false discovery rate (FDR) < 0.01 was regarded as differentially expressed. Subsequently, the Gene Ontology (GO) functional enrichment and Kyoto Encyclopedia of Genes and Genomes (KEGG) pathway enrichment analyses of the DEGs were performed using DAVID tools (https://david.ncifcrf.gov/).

### Quantitative real-time PCR assays (qRT-PCR)

qRT**-**PCR was performed with the SYBR Green Supermix (Bio-Rad, Hercules, USA) according to standard protocols [44]. Housekeeping gene GAPDH was used as an internal control. The following primers were used for qRT-PCR: DDX17-F, 5′-GAACATCCGGAAGTAGCAAGG-3′, DDX17-R, 5′-GATCCATCAACACATCCATTACATAT-3′; SPINK6-F, 5′-GGCATGTTTCTGCTCCTCTC-3′, SPINK6-R, 5′-TTTTCCAGGATGCTTTAGGC-3′; MYL9-F, 5′-CACCAGAAGCCAAGATGTCC-3′, MYL9-R, 5′-TTGAAAGCCTCCTTAAACTCC-3′; SLFN11-F, 5′-CCTGGTTGTGGAACCATCTT-3′, SLFN11-R, 5′-CTCTCCTTCTCTTGGTCTCTCT-3′; MAGEA6-F, 5′-AAAGGCAGAAATGCTGGGGAG-3′, MAGEA6-R, 5′-AGGCAGGTGGCAAAGATGTACAC-3′; and GAPDH-F, 5′-GTCTCCTCTGACTTCAACAGCG-3′, GAPDH-R, 5′-ACCACCCTGTTGCTGTAGCCAA-3′.

### RNA immunoprecipitation (RIP)

To validate the interaction between DDX17 and the mRNA of the related genes, the Magna RIP kit (Millipore) was used. The purified RNA was isolated with TRIzol® Reagent (Invitrogen) and reverse transcribed, and the relative gene expression of MYL9 and MAGEA6 was measured by qPCR, which was performed as described above.

### Cell adhesion assay

Cells (1 × 10^4^ cells/ well) were seeded into 96-well plates which were precoated with Corning Matrigel matrix (Corning, NY, USA) at 1 mg/ml. Plates were incubated for 2 h at 37 °C, then non-adherent cells were removed by a gentle washing four times with PBS, and the remaining cells were fixed with 4% paraformaldehyde for 30 min and stained with 0.1% crystal violet for 15 min. After washing, images were acquired using an inverted fluorescence microscope.

### Accession numbers

Sequence data have been deposited into the GEO of the NCBI, with the series entry (https://www.ncbi.nlm.nih.gov/bioproject/PRJNA796695).

### Statistical analysis

All assays were performed in triplicate on three independent experiments. The data were first tested for normality using the Shapiro-Wilk test. The differences in various groups were analyzed for statistical significance by two-way analysis of variance (ANOVA). The significant differences between two groups were analyzed by two-tailed Student’s *t*-test or Mann–Whitney test using GraphPad Prism software. *p*-values < 0.05 were considered statistically significant and set as follows: *p < 0.05; **p < 0.01; ***p < 0.001.

## Supplementary information

Author Contribution Statement

Table S1

Supplementary Figures

Supplementary Table and Figure legends

original images of western blot files

## Data Availability

The datasets used and/or analyzed during the current study are available from the corresponding author on reasonable request.
