# Supplementary material for: DDX17 promotes the growth and metastasis of lung adenocarcinoma
Source: Cell Death Discov. 2022 Oct 22;8:425. doi: 10.1038/s41420-022-01215-x (PMC9588018; doi:10.1038/s41420-022-01215-x)
Supplement: Supplementary file 1 — Author Contribution Statement [file 41420_2022_1215_MOESM1_ESM.pdf]

DECLARATION OF CONTRIBUTIONS TO ARTICLE

ADMC

Manuscript Number: CDDISCOVERY-22-4794R	Journal Name: *Cell Death Discovery*	(the 'Journal')
Proposed Title of the Contribution: **DDX17 promotes the growth and metastasis of lung adenocarcinoma**		(the 'Contribution)
Author(s): Xiaohui Liu, Lu Li, Chengjie geng, Shiyuan Wen, Cuiqiong Zhang, Chunmiao Deng, Xuejuan Gao, Gong Zhang, Qing-yu He, Langxia Liu		(the 'Authors')

For all *CDD* articles, each person named as an author in the published version must be able to show he or she has contributed substantially to the article.

Authorship credit should be based on 1) substantial contributions to conception and design, acquisition of data, or analysis and interpretation of data; 2) drafting the article or revising it critically for important intellectual content; and 3) final approval of the version to be published. Authors should meet conditions 1, 2 and 3.

Any person who cannot be shown to have made a substantial contribution to the article cannot be listed as an author in the final version. The name of any person who is deemed to have made a minor contribution can, however, appear in the Acknowledgments section of the article.

Please complete the table below to indicate the contributions of all named authors to the manuscript.

Author Full Name:	Specification of Contribution to the Manuscript:
Xiaohui Liu	performed the tumor xenograft experiments and in vivo tumor metastatic assay, analyzed the data and drafted the paper.
Lu Li	performed MTT, Clone formation assays, immunofluorescence assays, qRT-PCR,RIP assays.
Chengjie Geng	performed cell adhesion assay, and created MAGEA6 and MYL9 constructs.
Shiyuan Wen	performed MTT and Clone formation assays, created the DDX17 knockdown H1299 and A549 cell lines.
Cuiqiong Zhang	performed transwell assay.
Chunmiao Deng	created DDX17 constructs.
Xuejuan Gao	supervised the study.
Gong Zhang	supervised the study, and drafted the paper.
Qing-yu He	supervised the study.
Langxia Liu	designed and supervised the study and drafted the paper.

Please complete the table below to indicate the contributions of all named authors to the figures.

Figure 1:

Xiaohui Liu performed the Cancer Genome Atlas (TCGA), Clinical Proteomic Tumor Analysis Consortium (CPTAC) data analysis (Fig.1 A-E). Lu Li performed western blot analysis (Fig. 1F).

Figure 2:

Lu Li performed western blot analysis(Fig. 2A). Shiyuan Wen performed MTT and Clone formation assays and analysis (Fig. 2B-C); Xiaohui Liu performed the tumor xenograft experiments and in vivotumor metastatic assay (Fig. 2D,E,I,J). Cuiqiong Zhang performed transwell assay (Fig. 2F,G,H).

Figure 3:

Xiaohui Liu performed RNA-Seq data analysis (Fig. 3B and Supplementary Fig. 1A-D). Lu Li performed qRT-PCR, western blot analysis (Fig.3C-G)

Figure 4:

Chengjie Geng performed cell adhesion assay and mRNA decay assays (Fig.4A,C and Fig. S4).Lu Li performed immunofluorescence assays (Fig. 4B, D, E). Xiaohui Liu analyzed the data (Fig. 4A, C, F).

Figure 5:

Lu Li performed western blot analysis, MTT and Clone formation assays, immunofluorescence assays. Xiaohui Liu performed data analysis.

Figure 6:

Signed for and on behalf of the Author(s): Langxia Liu

Print Name: [illegible]

Date: 2022-10-8
