# Supplementary figures and images for: DDX17 promotes the growth and metastasis of lung adenocarcinoma
Source: Cell Death Discov. 2022 Oct 22;8:425. doi: 10.1038/s41420-022-01215-x (PMC9588018; doi:10.1038/s41420-022-01215-x)

## Slide 1
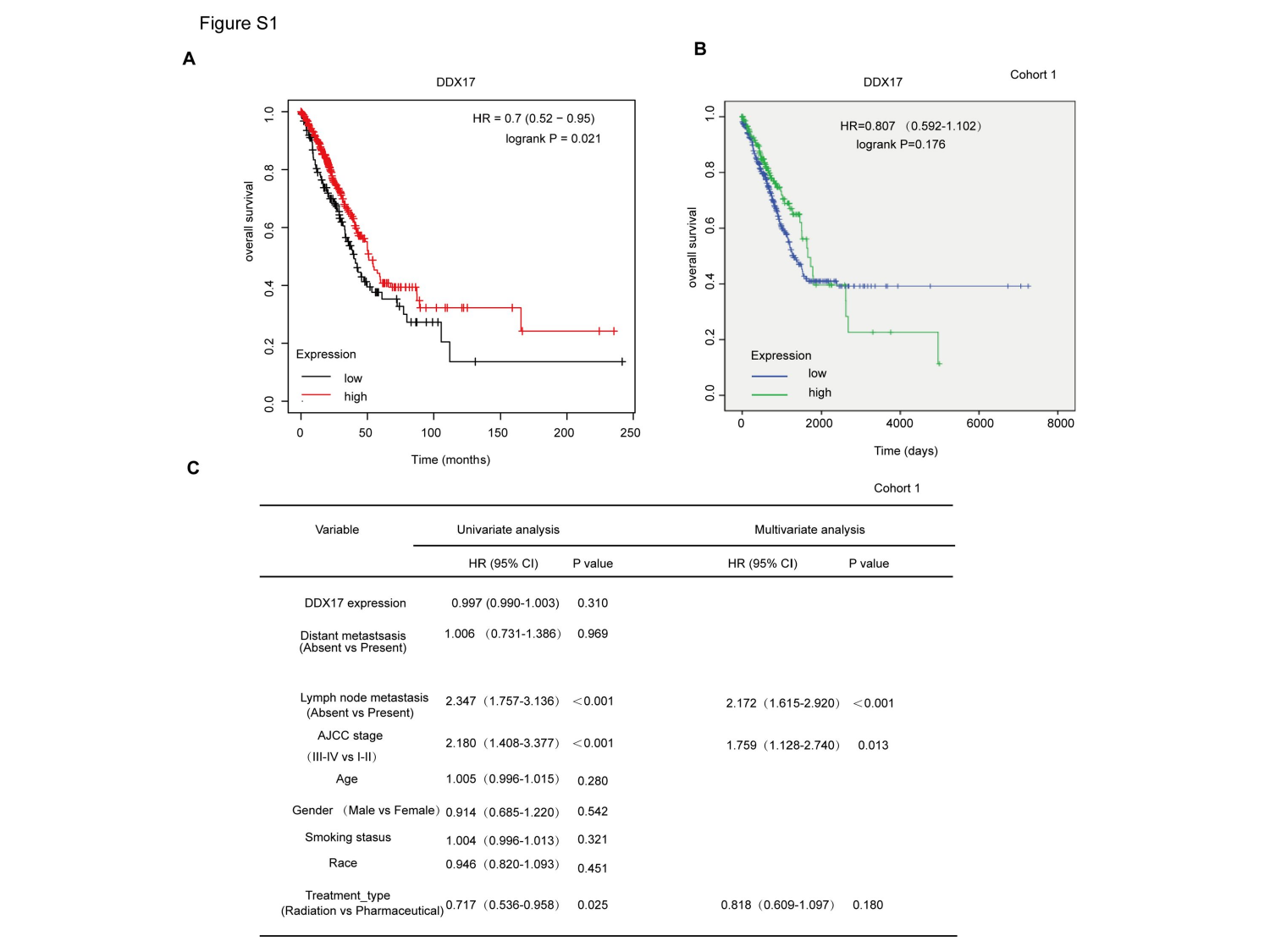

## Slide 2
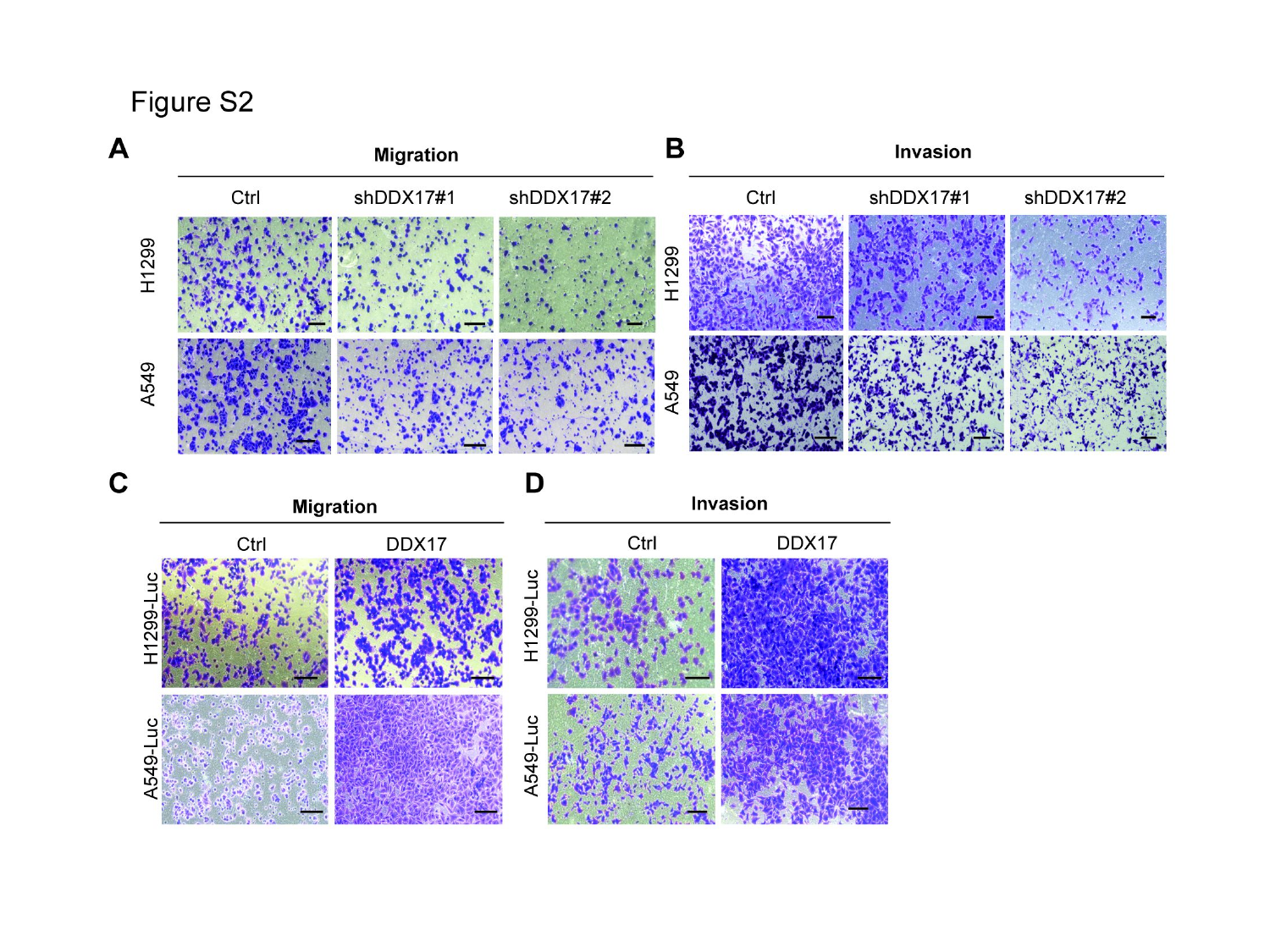

## Slide 3
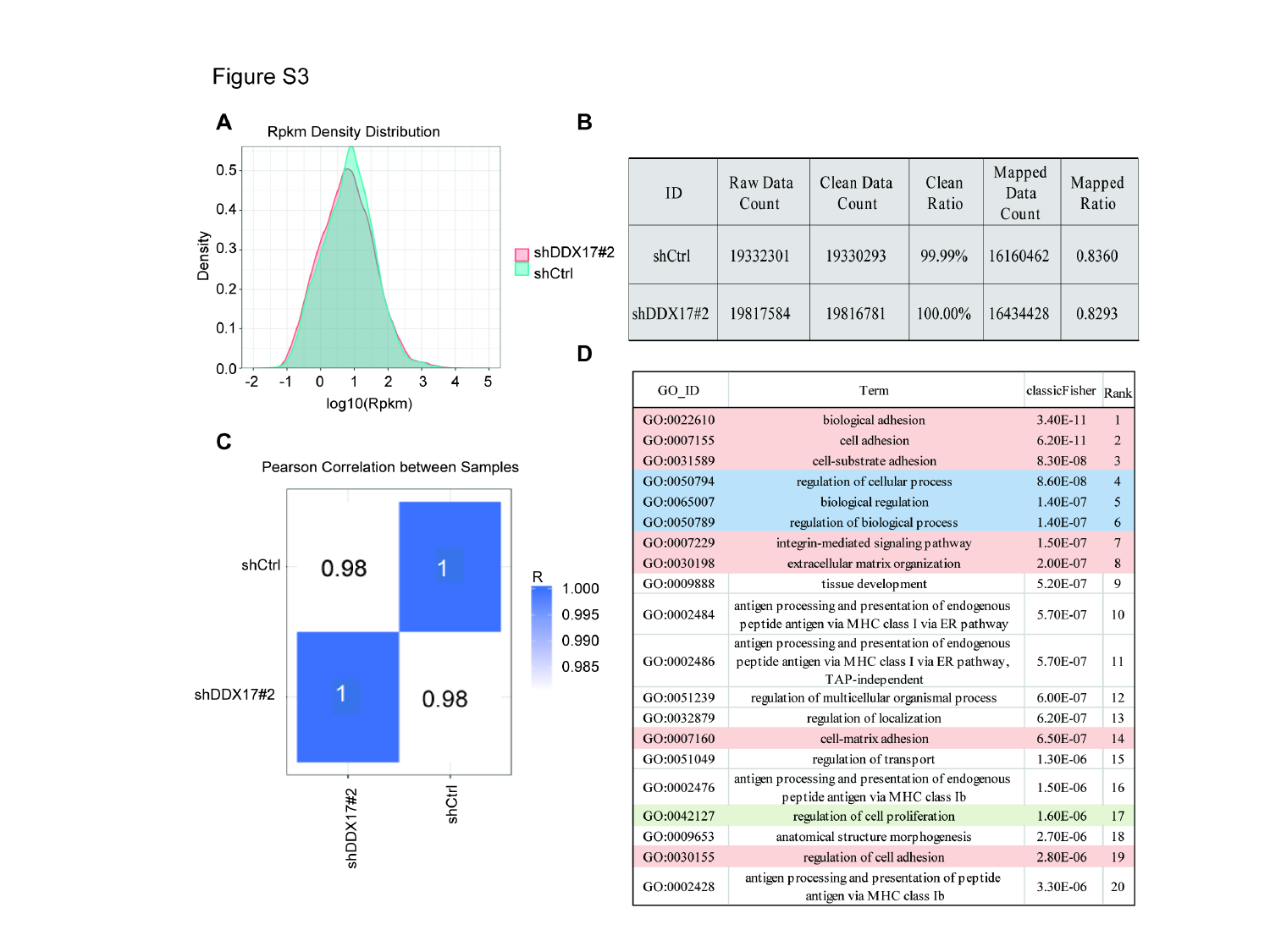

## Slide 4
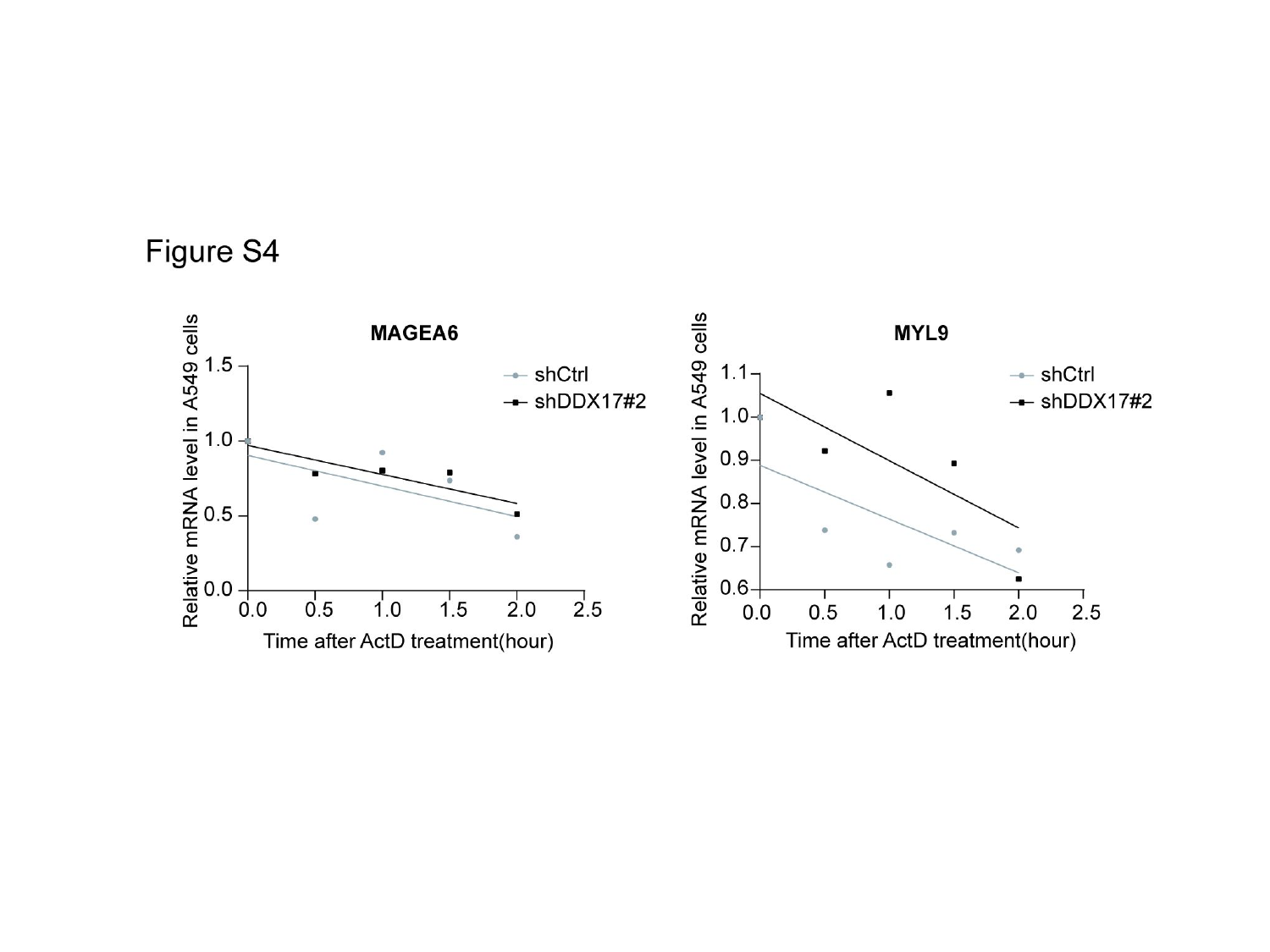

## Slide 5
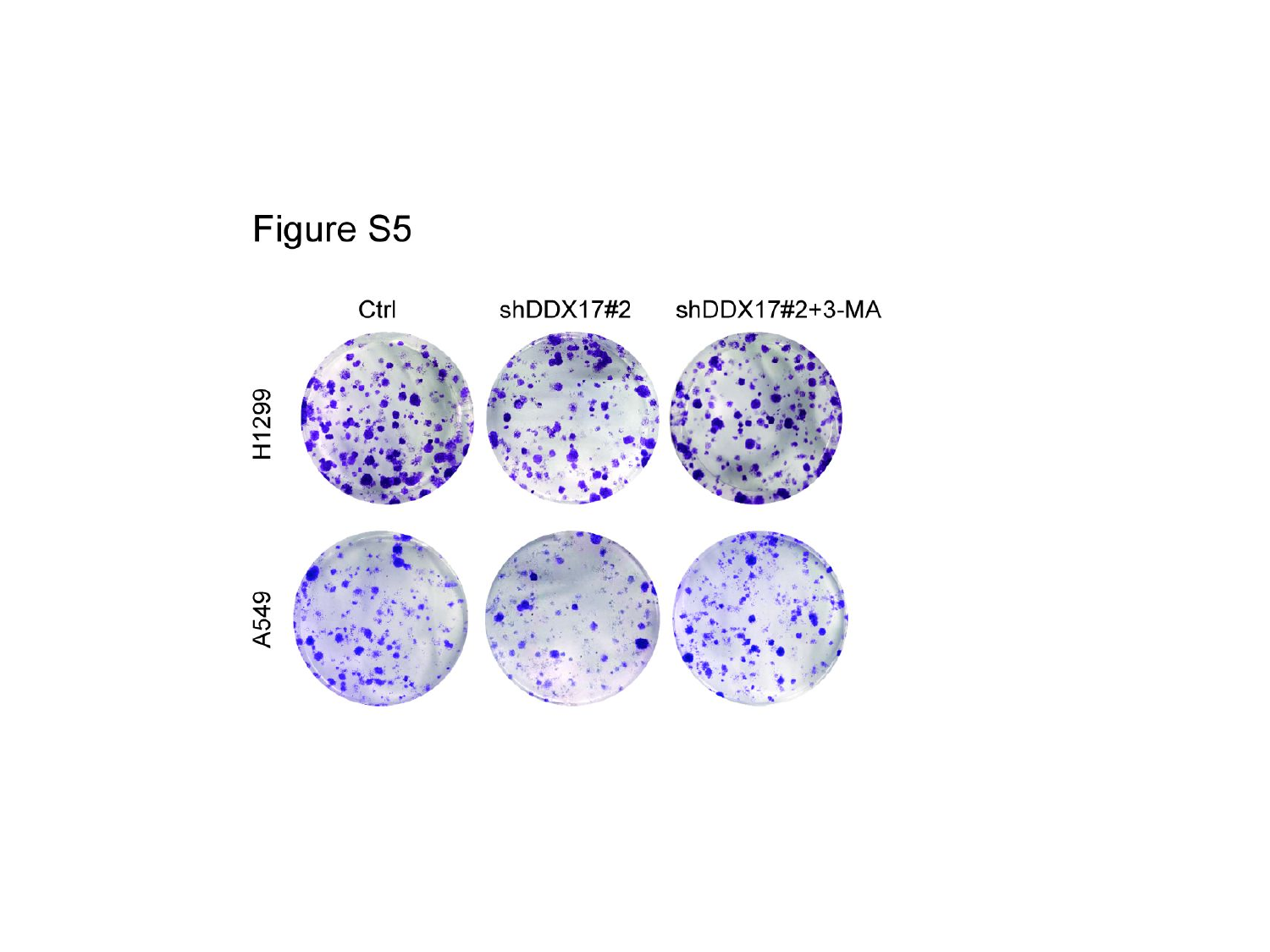

Supplement: Supplementary file 3 — Supplementary Figures [file 41420_2022_1215_MOESM3_ESM.ppt]
