# Supplementary material for: DDX17 promotes the growth and metastasis of lung adenocarcinoma
Source: Cell Death Discov. 2022 Oct 22;8:425. doi: 10.1038/s41420-022-01215-x (PMC9588018; doi:10.1038/s41420-022-01215-x)
Supplement: Supplementary file 4 — Supplementary Table and Figure legends [file 41420_2022_1215_MOESM4_ESM.doc]

**SUPPLEMENTARY TABLE AND FIGURE LEGENDS**

**Table S1 The expression of DDX17 in the clinical samples from TCGA database.**

The mRNA expression level of DDX17 in 594 LUAD patients and the corresponding clinical information were obtained from TCGA database on April 1, 2021 (cohort 1).

**Fig. S1 Survival analysis of DDX17 in LUAD patients.**

**A**, Kaplan-Meier plotter analysis of the correlation between DDX17 level with overall survive in LUAD patients (https://kmplot.com/analysis/, n=513). **B**, Survival analysis was conducted between LUAD patients with expression levels of DDX17 in

cohort 1(obtained from TCGA database, n = 594). **C**, Univariate and multivariate analyses incohort 1.

**Fig. S2 DDX17 promotes LUAD cell growth and invasion *in vitro*.**

Cell migration (A) and invasion (B) abilities analyzed by transwell assays in DDX17-knockdown H1299 and A549 cells as indicated. Transwell migration (C) and invasion (D) assays were performed in H1299-Luc and A549-Luc with stable

DDX17 overexpression, or control cells. scale bar = 100 μm.

**Fig. S3 Quantification of sequence data and GO enrichment analysis.**

**A** Density distribution Rpkm values. Densities were calculated using log_10_ Rpkm values based on non-normalized reads. (Green: shDDX17#2 cells; red: shCtrl cells; Rpkm: Reads Per Kilo bases per Million reads. **B** FANse3 ultra-high precision sequence alignment algorithm was used to compare the reads sequenced from each sample with the reference sequence (GCF_000001405.39_GRCH38.p13_RNA). **C** Pearson's correlation coefficient of RNA samples. **D** the functions of DEGs were predicted by analysis of GO by DAVID (<https://david.ncifcrf.gov/>).

**Fig. S4**  **DDX17 knockdown did not seem to affect the rate of decay of MAGEA6 or MYL9 mRNA.**

DDX17 silencing or their control cells were treated with actinomycin D (2.5μg/ml) for the indicated times. MAGEA6 and MYL9 mRNA levels were measured by qPCR.

**Fig. S5 3-methyladenine (3-MA) could partially restore the colony-forming capacity of DDX17-deficient cells.**

Clone formation assays were performed in DDX17 silencing and their control H1299 cells (top) treated with or without 3-MA (1mM), and A549 cells (bottom) treated with or without 3-MA (0.25mM), respectively.
