# Supplementary material for: DDX17 promotes the growth and metastasis of lung adenocarcinoma
Source: Cell Death Discov. 2022 Oct 22;8:425. doi: 10.1038/s41420-022-01215-x (PMC9588018; doi:10.1038/s41420-022-01215-x)

Original western blots of the Figure 1D

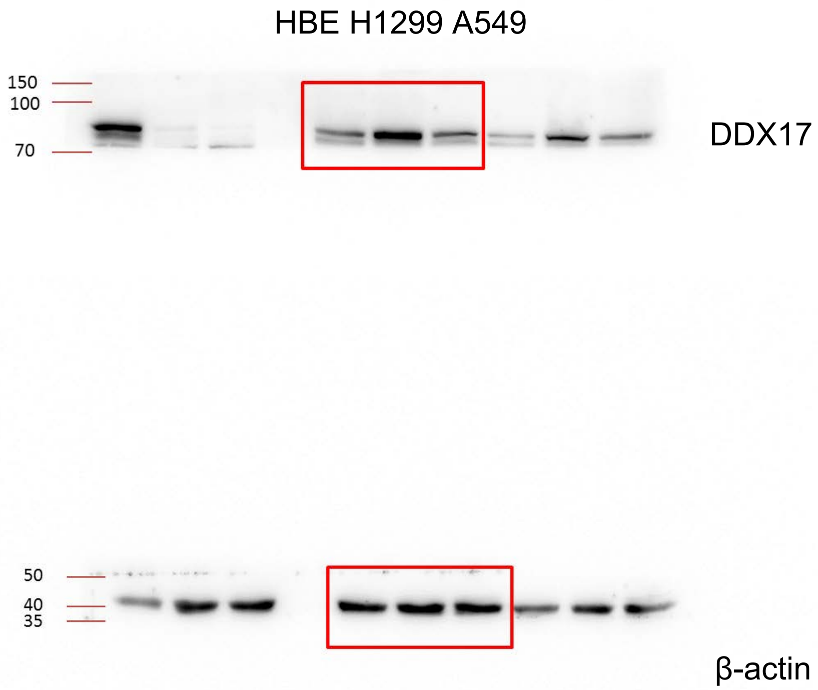

Original western blots of the Figure 2A

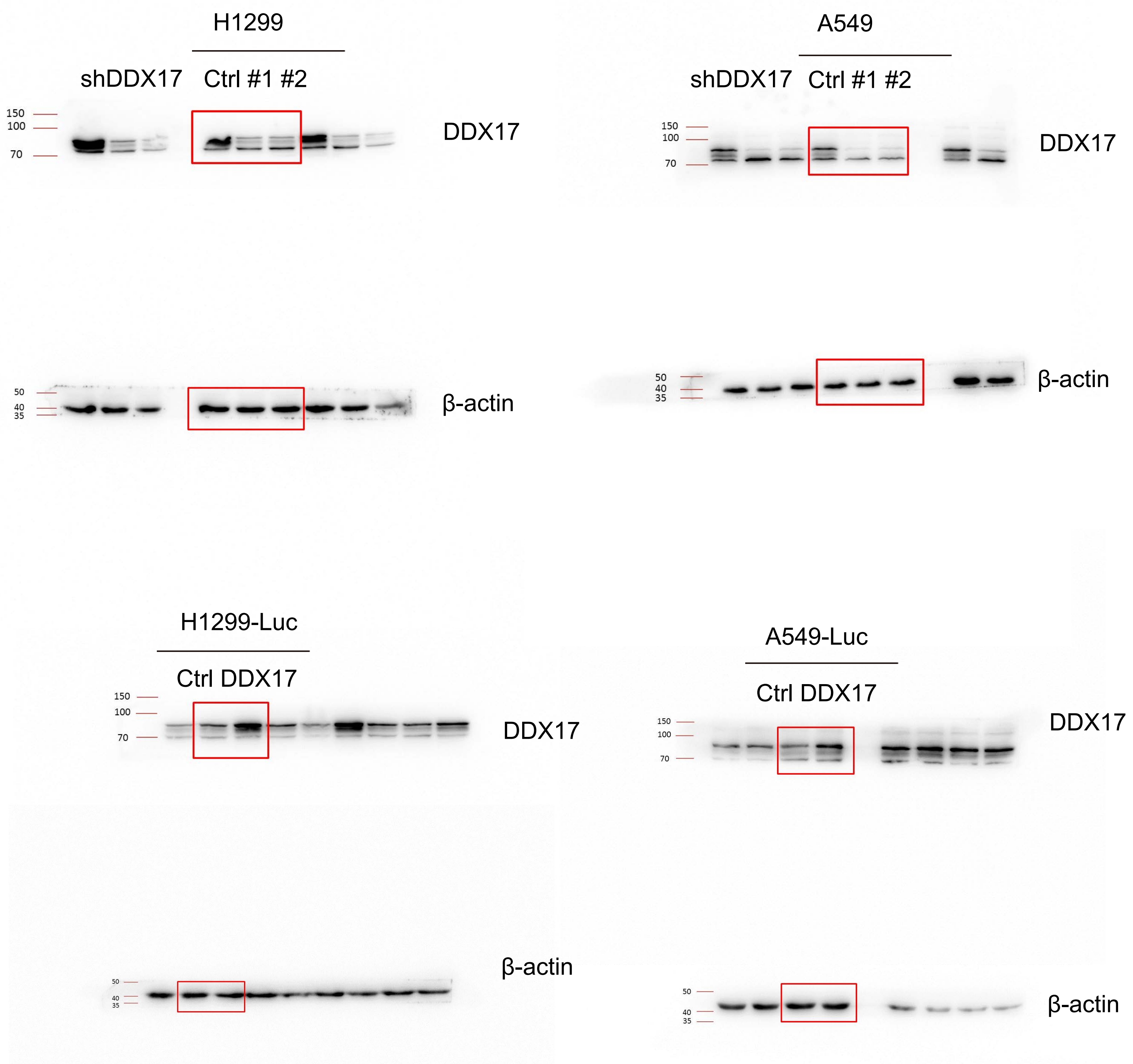

Original western blots of the Figure 3E

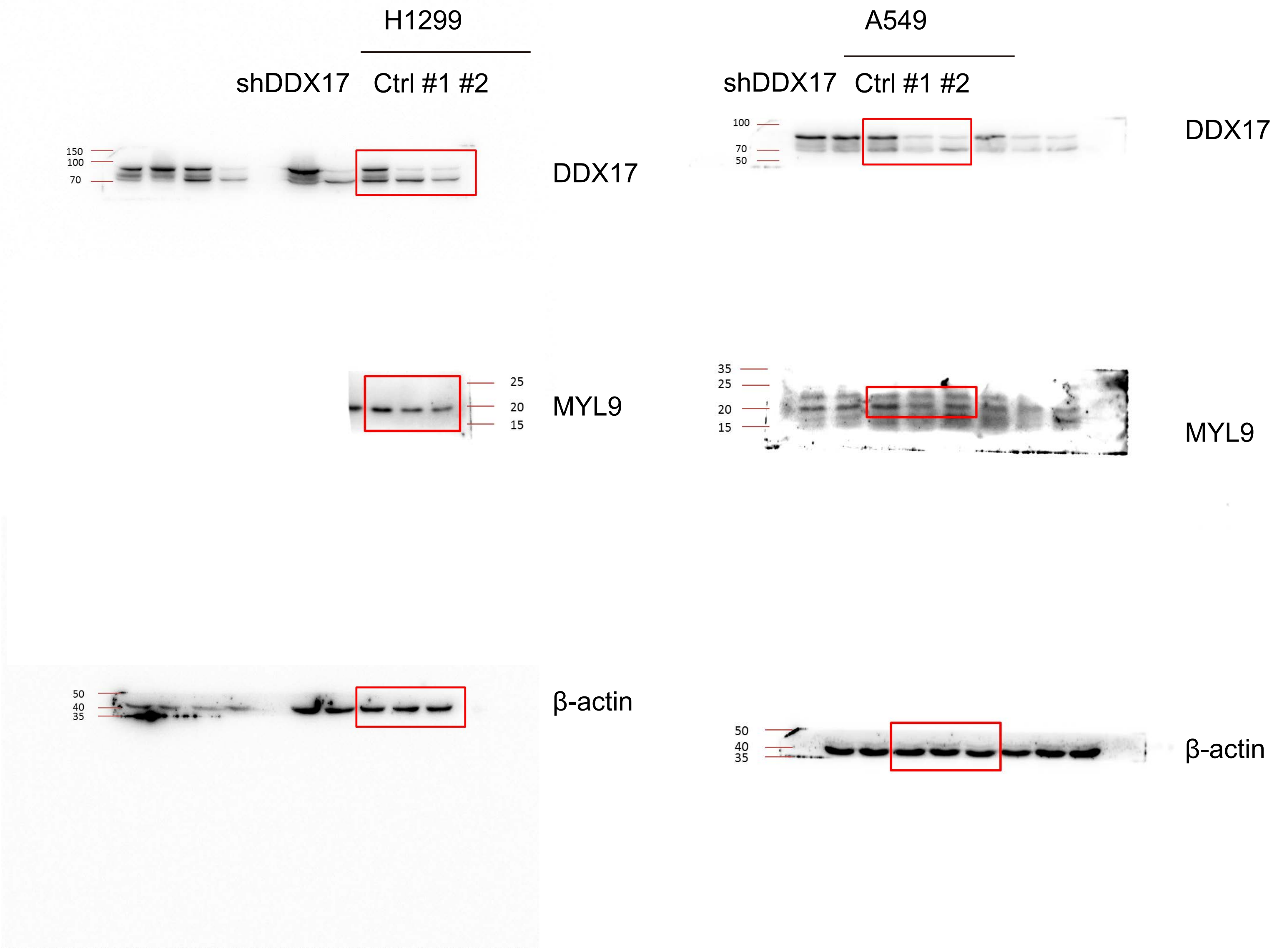

Original western blots of the Figure 3F

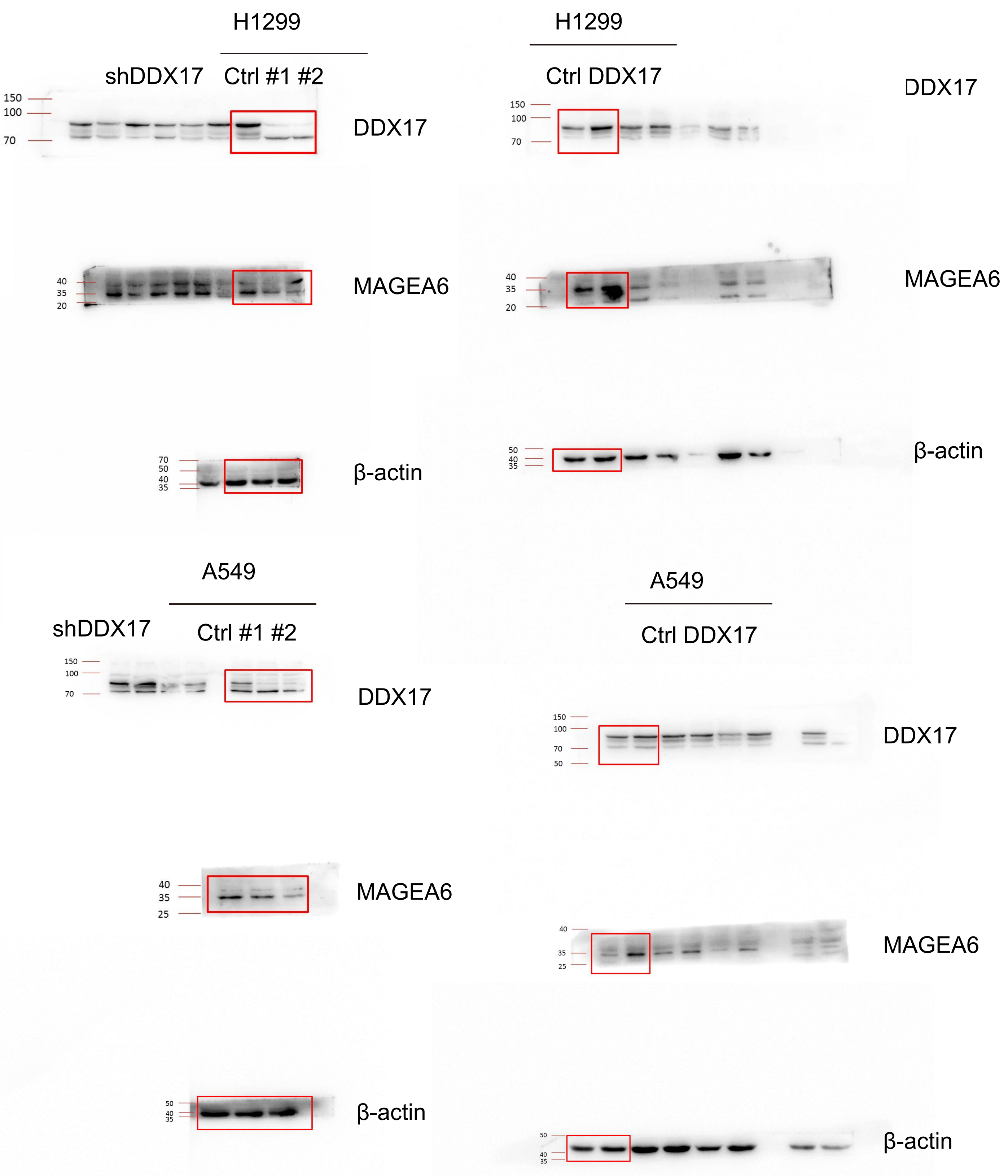

Original western blots of the Figure 4C

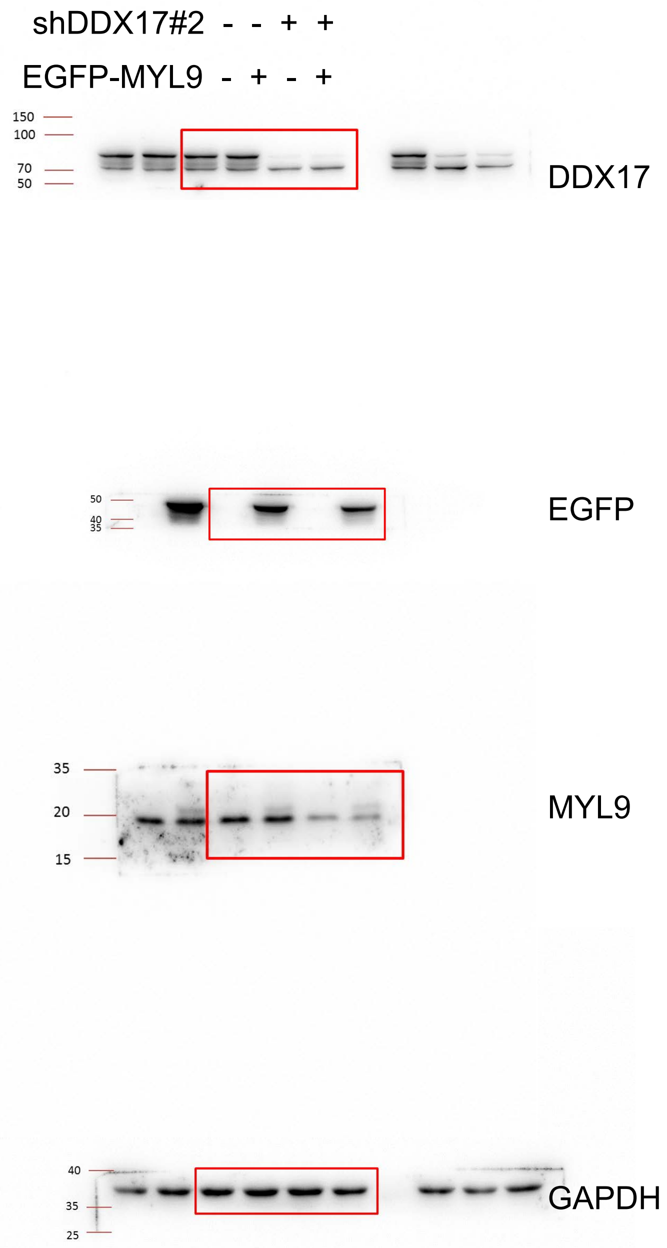

Original western blots of the Figure 5A

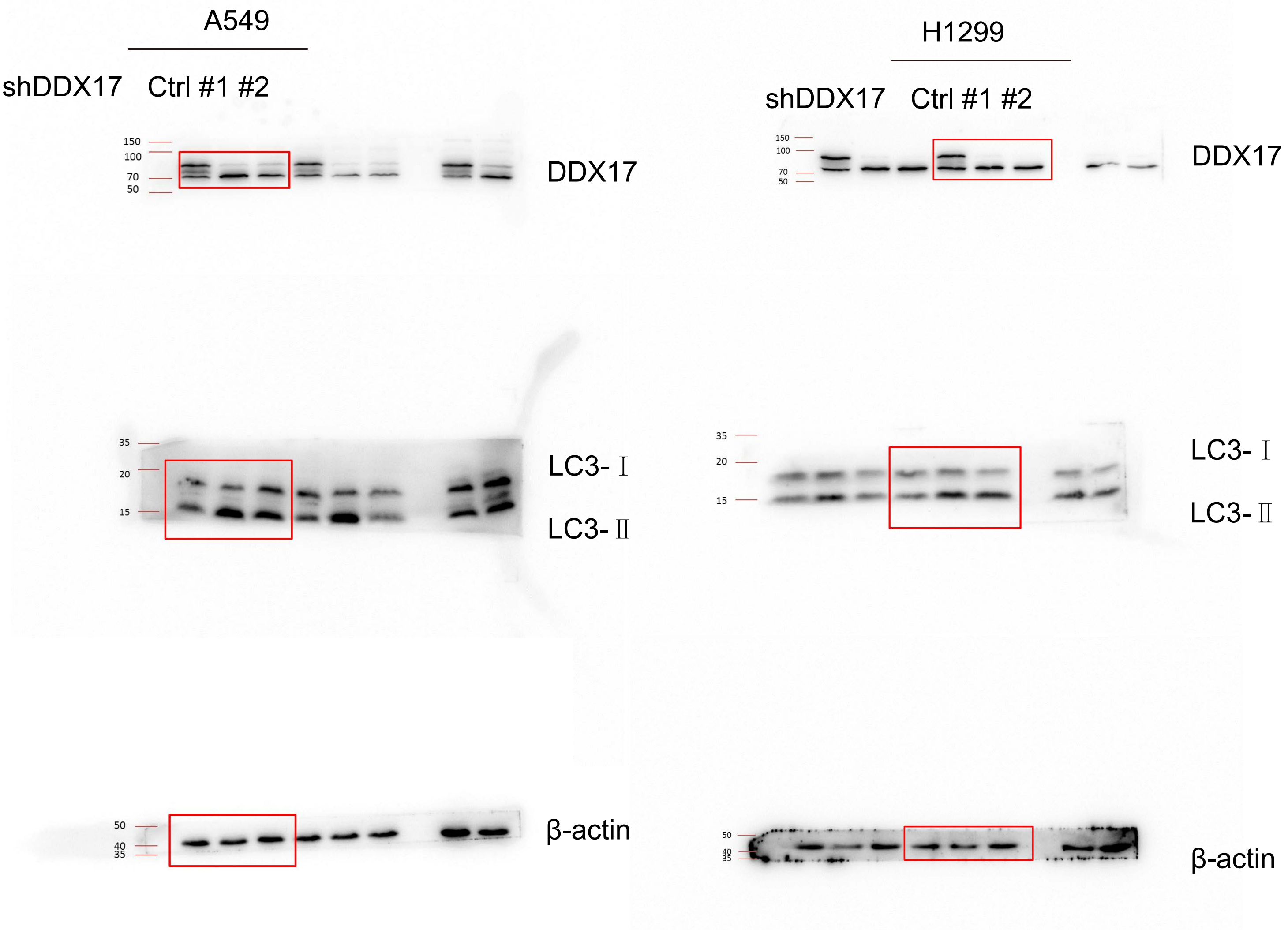

Original western blots of the Figure 5B

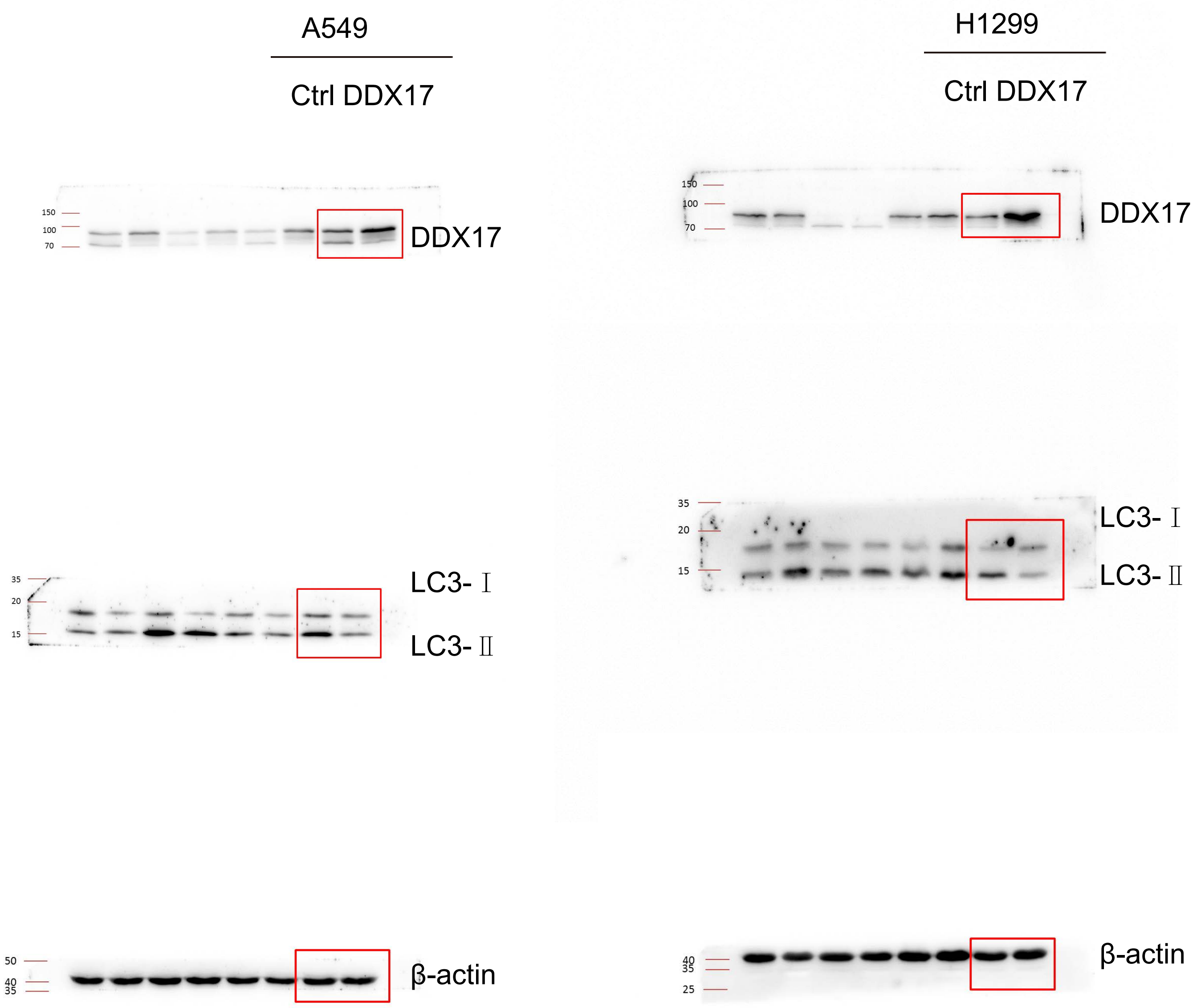

Original western blots of the Figure 5F left

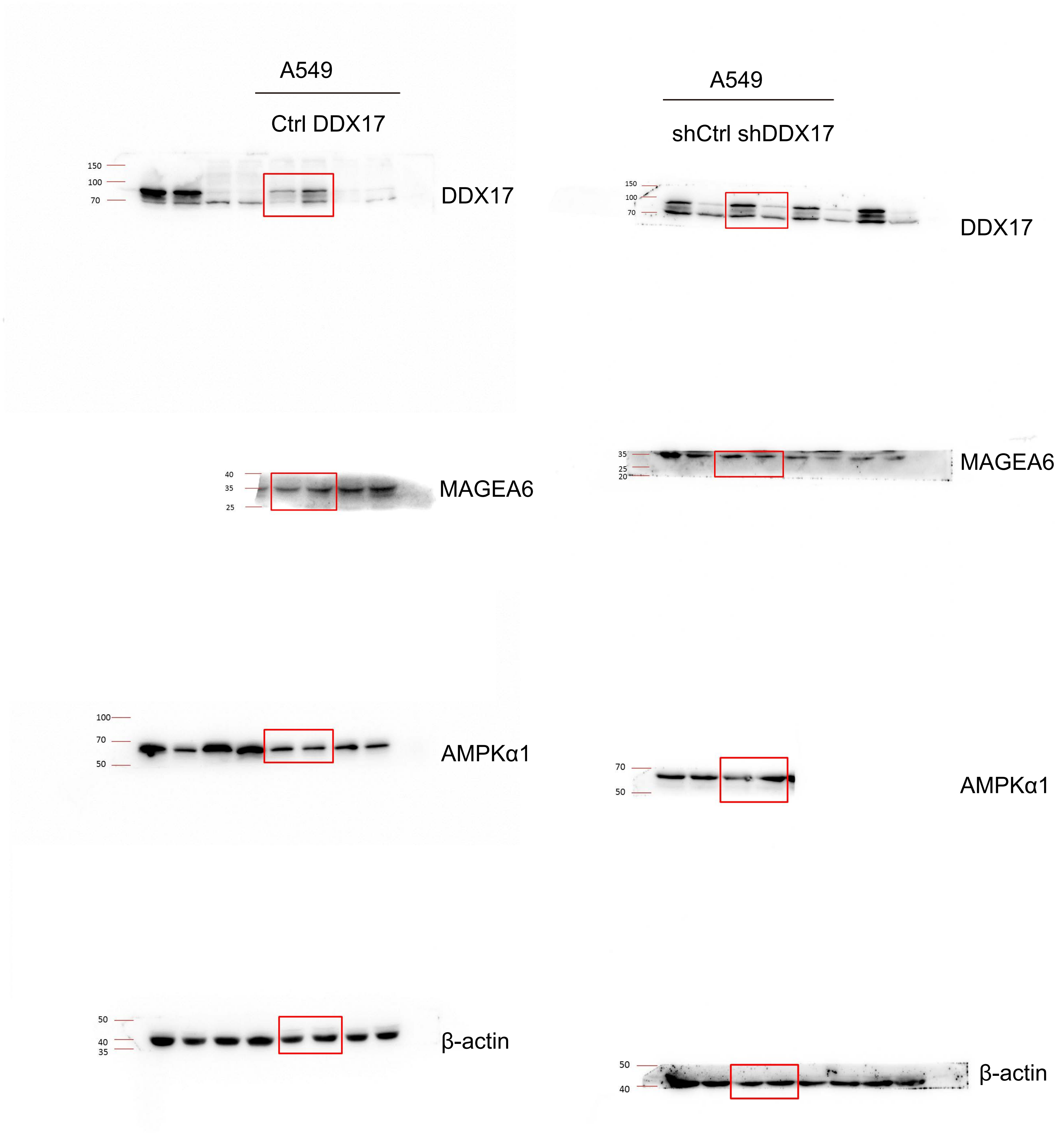

Original western blots of the Figure 5F right

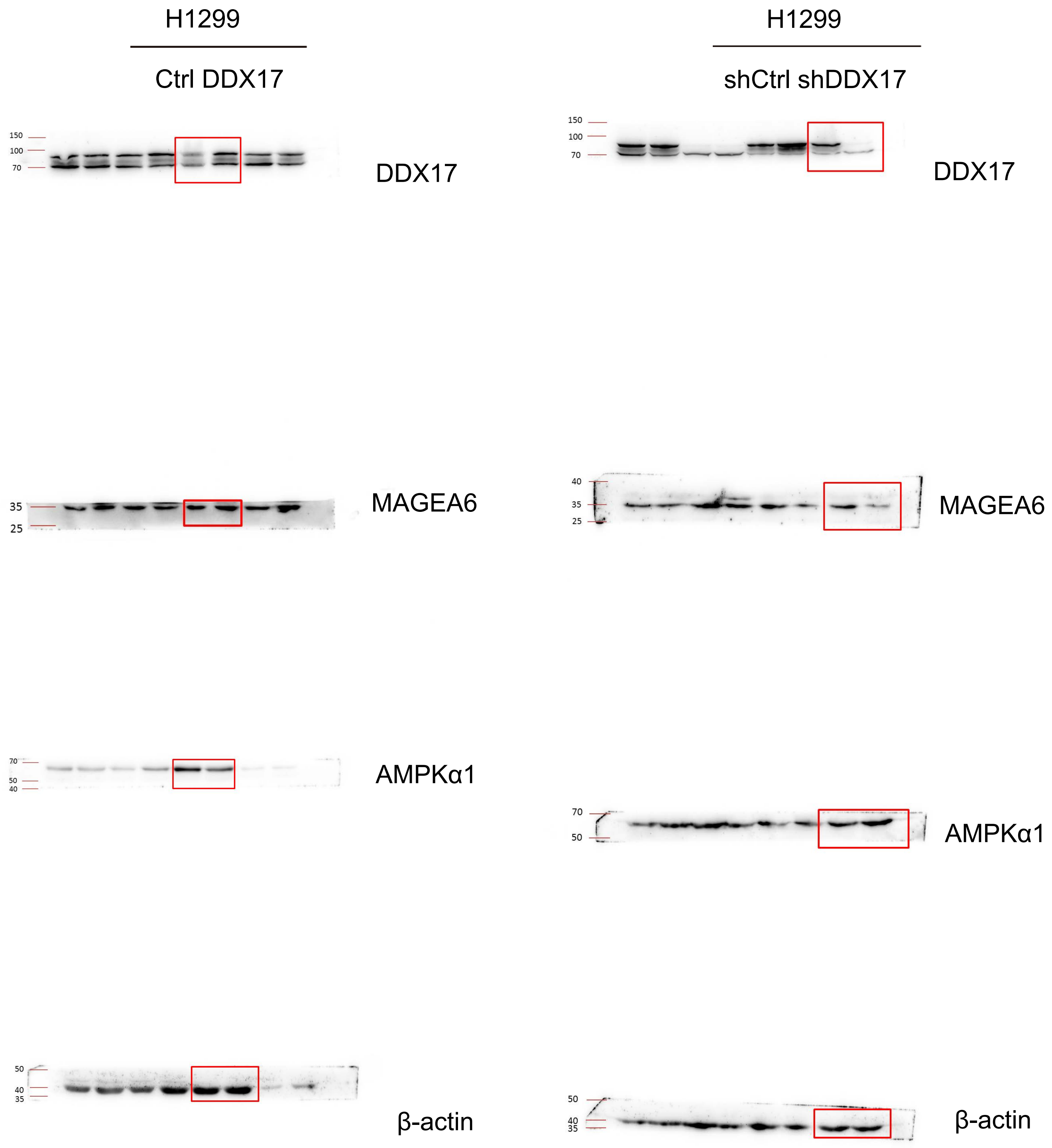

Original western blots of the Figure 5G

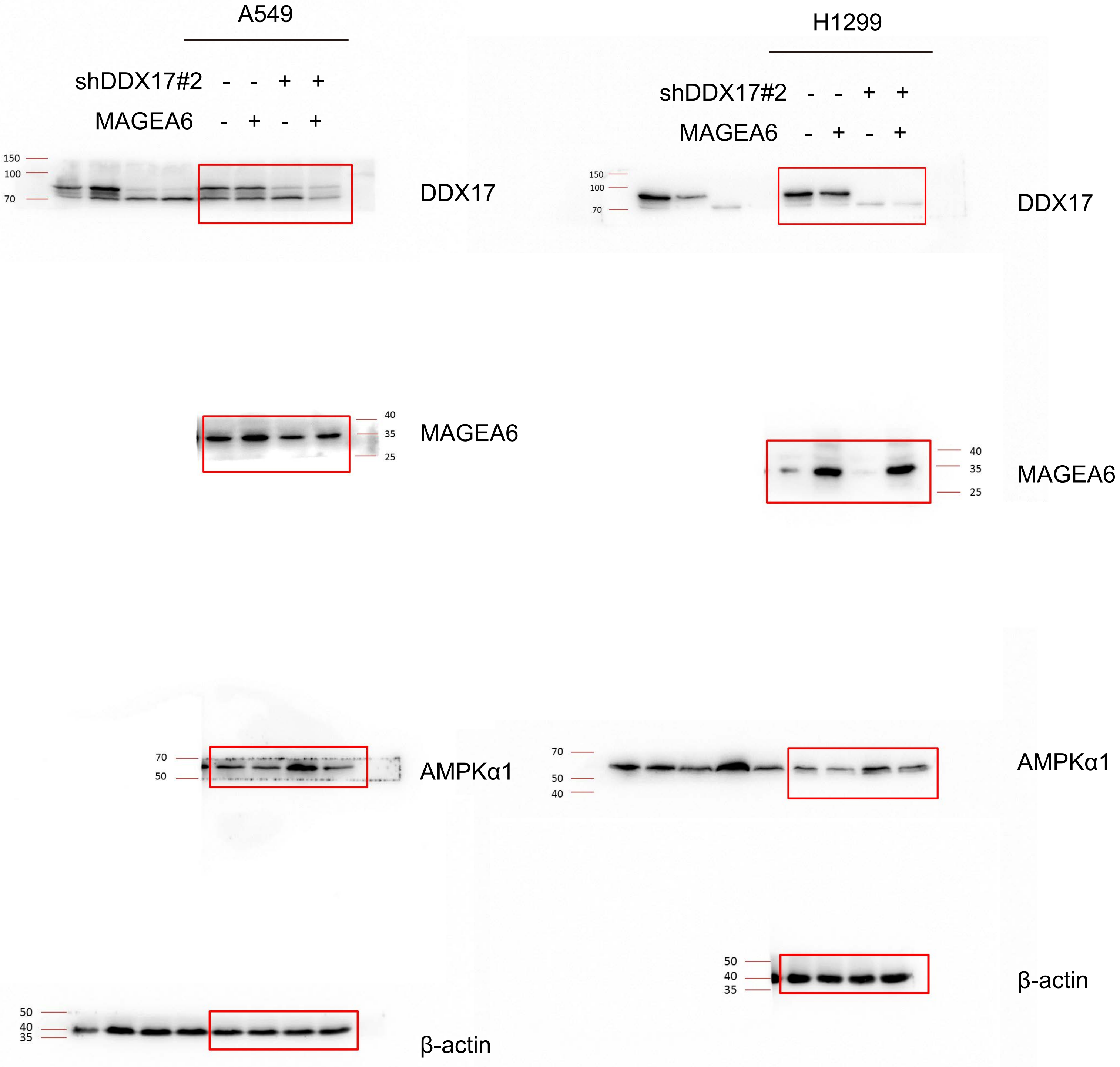

Original western blots of the Figure 5H

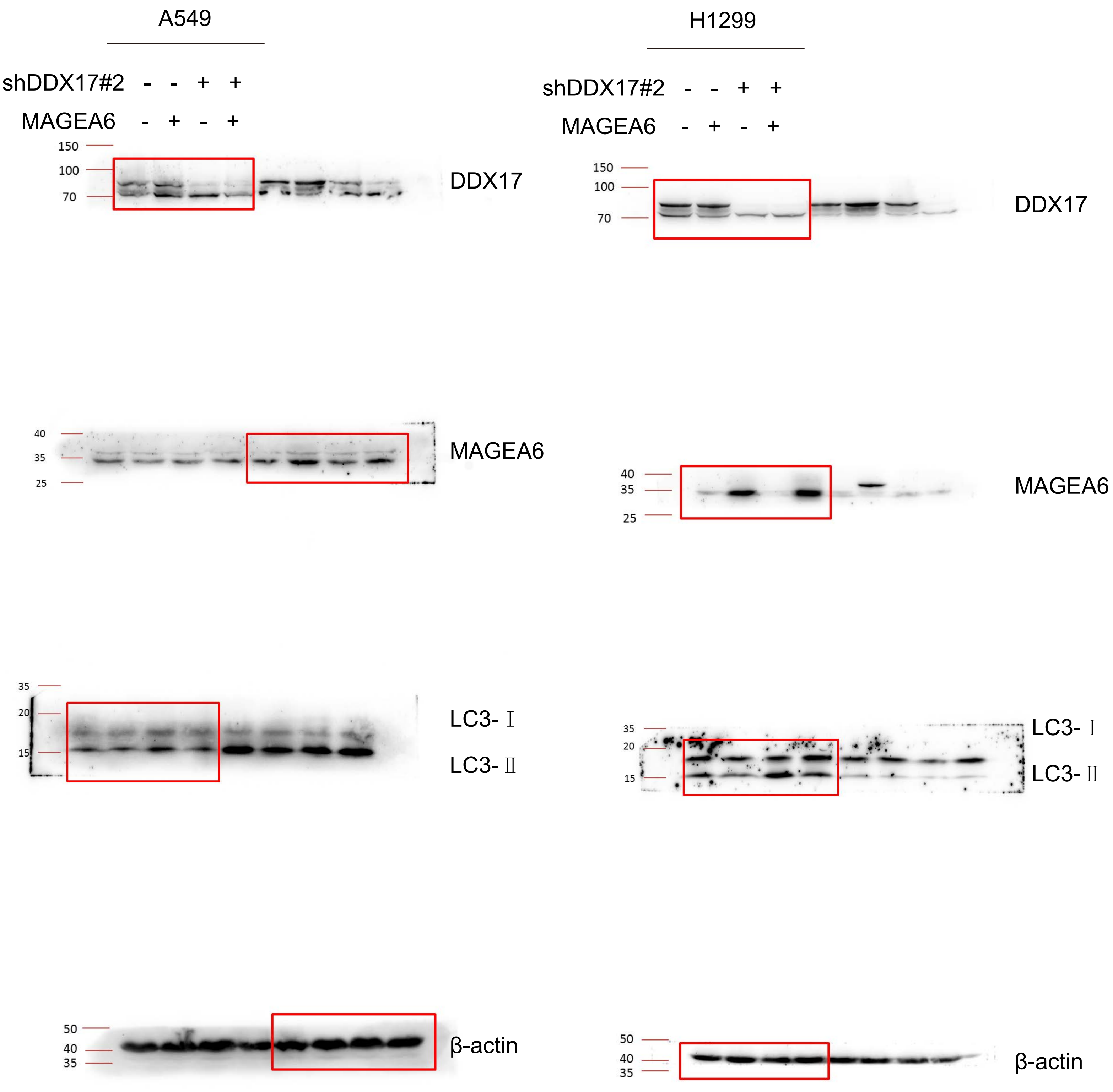

Supplement: Supplementary file 5 — original images of western blot files [file 41420_2022_1215_MOESM5_ESM.pdf]
